# Metformin and Probiotics Interplay in Amelioration of Ethanol-Induced Oxidative Stress and Inflammatory Response in an *In Vitro* and *In Vivo* Model of Hepatic Injury

**DOI:** 10.1155/2021/6636152

**Published:** 2021-04-15

**Authors:** Farhin Patel, Kirti Parwani, Dhara Patel, Palash Mandal

**Affiliations:** Department of Biological Sciences, P. D. Patel Institute of Applied Sciences, Charotar University of Science and Technology, Changa, 388421 Anand, Gujarat, India

## Abstract

Alcohol-induced liver injury implicates inflammation and oxidative stress as important mediators. Despite rigorous research, there is still no Food and Drug Administration (FDA) approved therapies for any stage of alcoholic liver disease (ALD). Interestingly, metformin (Met) and several probiotic strains possess the potential of inhibiting alcoholic liver injury. Therefore, we investigated the effectiveness of combination therapy using a mixture of eight strains of lactic acid-producing bacteria, commercialized as Visbiome® (V) and Met in preventing the ethanol-induced hepatic injury using *in vitro* and *in vivo* models. Human HepG2 cells and male Wistar rats were exposed to ethanol and simultaneously treated with probiotic V or Met alone as well as in combination. Endoplasmic reticulum (ER) stress markers, inflammatory markers, lipid metabolism, reactive oxygen species (ROS) production, and oxidative stress were evaluated, using qRT-PCR, Oil red O staining, fluorimetry, and HPLC. *In vitro*, probiotic V and Met in combination prevented ethanol-induced cellular injury, ER stress, oxidative stress, and regulated lipid metabolism as well as inflammatory response in HepG2 cells. Probiotic V and Met also promoted macrophage polarization towards the M2 phenotype in ethanol-exposed RAW 264.7 macrophage cells. *In vivo*, combined administration of probiotic V and Met ameliorated the histopathological changes, inflammatory response, hepatic markers (liver enzymes), and lipid metabolism induced by ethanol. It also improved the antioxidant markers (HO-1 and Nrf-2), as seen by their protein levels in both HepG2 cells as well as liver tissue using ELISA. Hence, probiotic V may act, in addition to the Met, as an effective preventive treatment against ethanol-induced hepatic injury.

## 1. Introduction

Alcohol-induced hepatic injury is the main root of morbidity and mortality universally among individuals who misuse alcohol [1]. Chronic alcohol consumption eventually leads to ALD, which encompasses steatosis, alcoholic hepatitis, fibrosis, cirrhosis, and lately, hepatocellular carcinoma (HCC) [2]. Several studies reported that oxidative stress and inflammatory responses play a key role in the development and progression of alcohol-induced liver damage [3, 4]. A few of the reasons behind the progression are (a) expression of pro-inflammatory enzymes, cytokines, and chemokines through Kupffer cell activation [5] and (b) overproduction of reactive oxygen species (ROS) leading to ROS-mediated liver injury [6], and this can eventually lead to oxidative stress (excessive ROS generation leads to increase oxidant formation and reduced levels of antioxidants) [7] and also lipid peroxidation (increased ROS production leads to the generation of 4-HNE which forms adducts with protein and DNA) [8]. Thus, inhibiting the inflammation and oxidative stress would play a major role in preventing ethanol-induced hepatic injury. Till the time, limited acceptable developments have been completed in controlling the progression of liver injury. Thus, unique and trustworthy therapeutic approaches are strongly needed.

Metformin (Met) is a commonly approved antihyperglycemic drug of the biguanide family commonly used to control type 2 diabetes. In humans, there is a resilient connection between hepatic insulin resistance and steatohepatitis as well as fatty liver. In many animal and human studies, Met has shown protection in NASH and nonalcoholic fatty liver disease (NAFLD). In NAFLD patients, Met treatment shows decreased liver dysfunction [9]. The accepted history of NAFLD presents similarities to that of ALD. Certain findings have revealed that fatty liver disease, either alcoholic or nonalcoholic, showed alike mechanisms and pathological characteristics. Certain studies showed that the gut microbiota might play a crucial role in Met's therapeutic effect, elucidating the fact that the gastrointestinal tract (GIT) is an important site in the Met action [10–12]. It is even appreciable that improving the therapeutic effect of Met using gut microbiome modulators like probiotics may reduce oxidative stress and ER stress and increase the level of the anti-inflammatory cytokine.

On the other hand, over the last two decades, in food and feed industries along with clinical and medical fields, probiotic application has increased due to their various functional capabilities. Probiotics are defined as monocultures or assorted cultures of microorganisms that can be adequately administered to improve the functional properties of the gut microbiota [13]. A very recent study showed that the cell-free supernatant obtained from different strains of probiotics, namely, *Lactobacillus acidophilus*, *Lactobacillus bulgaricus*, *Streptococcus thermophilus*, *Bifidobacterium longum*, and *Lactobacillus plantarum*, had shown to possess the antioxidant potential and be able to scavenge the superoxide radicals and hydroxyl radicals. These are the radicals that would lead to oxidative stress and therefore upregulation of pro-inflammatory cytokines. The in vitro mechanism of action of probiotics could partially be explained by its antioxidant potential, which reduced the oxidative stress and further damage associated with it [14, 15]. There are reports which suggest that probiotics release certain cellular components (which could be also a part of cell-free lysate), which may cause immunomodulation, by regulating the activation of immunological cells [16]. Probiotic Visbiome® (V) (ExeGi Pharma, LLC, Rockville, MD) (original De Simone Formulation) is a mixture of eight strains of lactic acid-producing bacteria (*Lactobacillus acidophilus*, *Lactobacillus paracasei*, *Lactobacillus delbrueckii* subspecies *bulgaricus*, *Lactobacillus plantarum*, *Bifidobacterium longum*, *Bifidobacterium infantis*, *Bifidobacterium breve*, and *Streptococcus salivarius* subspecies *thermophilus*). Probiotic V contains the same formulation found in VSL#3 (sold under VSL Pharmaceuticals, Inc.) produced before January 31, 2016. In alcohol-induced intestinal barrier injury, the VSL#3/heat-killed VSL#3 alone or together with glutamine is proven to be effective in reducing the intestinal permeability and upregulating the tight junction protein expression, thus preventing the entry of endotoxins and other bacterial products into the portal circulation and therefore results in downregulating the TNF-*α* expression [17]. Treatment with VSL#3 for 3 months in alcoholic liver cirrhosis patients (AC) groups (*n* = 10) significantly decreased plasma levels of alanine aminotransferase (ALT), aspartate aminotransferase (AST), and gamma-glutamyl transferase (GGT) and decreased the levels of MDA and 4-HNE, whereas plasma cytokines like TNF-*α* and IL-6 were reduced along with an increase in IL-10 levels [18].

Administration of probiotics along with Met can help in maintaining gut integrity along with improving the efficacy of the Met, as recent studies have proven that treatment with *Akkermansia* spp. improved glucose homeostasis, suggestive of better efficacy of Met via gut modulation [10]. Various reports suggesting the beneficial effects of combination therapy with probiotic and Met in many diseases including diabetes mellitus, colorectal cancer, and nonalcoholic steatohepatitis (NASH) exist [12, 19, 20], but there have been narrow efforts to evaluate the possible benefits of the combined administration of probiotics and Met in ethanol-induced hepatic injury. Based on the above studies as well as the individual protective role of probiotics and Met towards alcoholic liver injury, the current study evaluated the combined effect of probiotic V and Met in modulating ethanol-induced hepatic injury using *in vitro* and *in vivo* models.

## 2. Materials and Methods

Entirely each chemical/reagent was acquired from Himedia Laboratories (India), except specified. Molecular grade ethanol (99.8% purity) (Himedia Laboratories, India) was purchased for the induction of hepatic injury. Met was purchased from Sigma-Aldrich (USA). DMEM (Dulbecco's Modified Eagle Medium) and RPMI-1640 were purchased from Gibco (USA). Fetal bovine serum (FBS) was purchased from Invitrogen (USA). TRIzol reagent, DCFDA, DNase-I, cDNA synthesis kit, and SYBR Green master mix were purchased from Thermo Fisher Scientific (USA). All the primer sequences were obtained from Sigma-Aldrich (USA). All purchased chemicals used in the experiments were of high molecular biology grade.

### 2.1. Cell Culture

In the current study, we used HepG2 cells and RAW 264.7 cells, acquired from National Centre for Cell Sciences (NCCS), Pune, India. In brief, HepG2 cells were cultured and maintained in DMEM [21] and RAW 264.7 cells were cultured in RPMI-1640 medium [22] supplemented with 10% FBS and 1% antibiotic-antimycotic solution in a humidified atmosphere of 5% CO_2_ at 37°C.

Throughout the experiment, the complete culture medium was removed and changed every other day for 3–4 days. The passage used for further experiments was between 20 and 35. After attaining 70% confluency, HepG2 cells and RAW 264.7 cells were maintained in respective serum-free medium overnight and incubated with ethanol alone as well as with probiotic V and Met alone or in combination as probiotic V and Met in the presence of ethanol.

### 2.2. Preparation of Bacterial Lysate Using Probiotic Visbiome®

Visbiome® (Lot #07197721) is a complex probiotic consortium comprising 112.5 × 10^9^ CFU/capsule of three viable lyophilized bacterial species: four strains of lactobacilli (*Lactobacillus acidophilus*, *Lactobacillus paracasei*, *Lactobacillus delbrueckii* subspecies *bulgaricus*, and *Lactobacillus plantarum*), three strains of bifidobacteria (*Bifidobacterium longum*, *Bifidobacterium infantis*, and *Bifidobacterium breve*), and *Streptococcus salivarius* subspecies *thermophilus*. Probiotic Visbiome® (V) containing 1 g of each stock was suspended in De Man, Rogosa, and Sharpe agar (MRS) broth to activate the culture. Overnight activated culture of probiotic V was centrifuged at the speed of 6000 rpm for 10 min (4°C). After centrifugation, both the cultures were washed twice with sterile PBS and resuspended at a final concentration of 10^8^ CFU/ml in MRS broth.

The culture of probiotic V at their respective final concentrations was sonicated for 30 min (repeating 10 s of sonication and 10 s of hold) with a sonicator. The bacterial cultures were centrifuged (1500 ×·g for 10 min), and individual supernatant (whole-cell extract) was obtained. Furthermore, the supernatant containing the whole-cell extract was centrifuged (6500 × g for 30 min) yielding cell cytosol (supernatant) and membrane (pellet). Bacterial cell disruption was confirmed by measuring the optical density (O.D.) of every sample at 600 nm after each sonication until the O.D. reached a persistent value. Protein concentration in each sample was measured using a Bradford assay [23]. The total protein concentration was found to be between 4 and 6 mg/ml in all bacterial lysates. The total cellular fluid of probiotic V was then suspended in DMEM/RPMI-1640 at suitable concentrations and further kept at −20°C for experiments.

### 2.3. Cell Viability Assay

**Figure 1:**
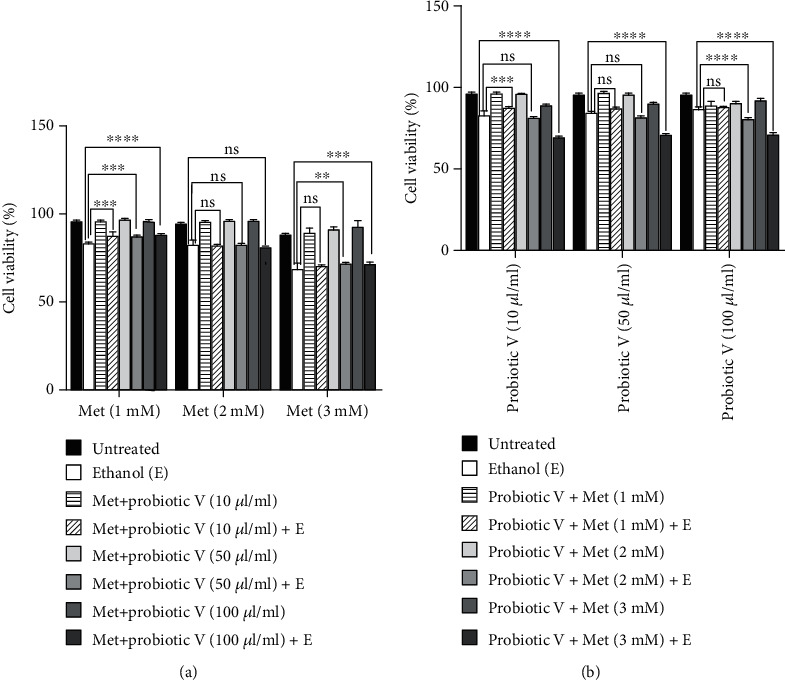
Effect of probiotic V and Met on the viability of HepG2 cells. (a) HepG2 cells were exposed to 100 mM ethanol and cotreated with probiotic V (10, 50, and 100 *μ*l/ml) and Met (1, 2, and 3 mM) in different combinations and compared with the individual doses of Met 1, 2, and 3 mM alone in the presence of ethanol (ethanol column in the graph) for 48 h; (b) HepG2 cells were exposed to 100 mM ethanol and cotreated with probiotic V (10, 50, and 100 *μ*l/ml) and Met (1, 2, and 3 mM) in different combinations and compared with the individual doses of probiotic V (10, 50, and 100 *μ*l/ml) alone in the presence of ethanol (ethanol column in the graph) for 48 h. The average percentage of cell viability after respective treatment was analyzed through MTT assay. Values represent the mean ± SD of three individual experiments. Statistical significance was assessed by one-way ANOVA followed by Tukey post hoc test. Statistical analysis: ^####^*p* < 0.0001 compared with control group; ^∗∗^*p* < 0.01, ^∗∗∗^*p* < 0.001, and ^∗∗∗∗^*p* < 0.0001 compared with ethanol group as well as individual treatment of probiotic V (10, 50, and 100 *μ*l/ml) and Met (1, 2, and 3 mM) groups in different combinations; ns stands for nonsignificant.

In the present *in vitro* experiments, the HepG2 cell line was treated with the suspension of bacterial lysates at final concentrations of 10, 50, and 100 *μ*l/ml, corresponding to 1, 5, and 10 mg (lyophilized bacterial mass)/ml. Cells were supplemented with a total of the bacterial lysate (10, 50, and 100 *μ*l/ml) corresponding to 10^8^, 5 × 10^8^, and 10^9^ CFU/ml in the culture medium. For each experiment, fresh bacterial lysates of probiotic V and Met were prepared for experimental studies (Figure 1).

The concentration of ethanol was determined based on the literature survey [24]. 3-(4,5-dimethylthiazol-2-yl)-2,5-diphenyltetrazolium bromide (MTT) assay was used to determine the cell viability as described by Kema et al. [22]. In brief, 5 × 10^4^ cells were seeded in a 96-well plates. After the cells attained the desired confluency, they were exposed to different concentrations of probiotic V (10, 50, and 100 *μ*l/ml) and Met (1, 2, and 3 mM), individually and in combinatorial doses (10 *μ*l/ml probiotic V with 1 mM, 2 mM, and 3 mM Met; 50 *μ*l/ml probiotic V with 1 mM, 2 mM, and 3 mM Met; 100 *μ*l/ml probiotic V with 1 mM, 2 mM, and 3 mM Met) in the presence and absence of 100 mM ethanol for 48 h. After 48 h treatment, each well was incubated with 10 *μ*l of MTT solution (0.45 mg/ml: final concentration) and kept in the dark at 37°C in a 5% CO_2_ atmosphere for 3 h. After the incubation period was over to each well, 100 *μ*l of dimethyl sulfoxide (DMSO) was added to solvate the formazan crystals. To determine the cell viability, absorbance was measured at 570 nm for each sample and calculated as in Equation (1) as follows:

(1) $$\begin{array}{c} \text{Cell viability}\left(\%\right)=\frac{\text{As}}{\text{Ac}}\times 100. \end{array}$$

As and Ac represent the absorbance of sample treatment and control, respectively.

### 2.4. In Vitro Induction of Hepatic Injury Model Using Ethanol Stimulation with Simultaneous Probiotic Visbiome® and Metformin Administration

To induce hepatic injury, 1.8 × 10^5^ HepG2 cells were plated in 60 mm culture dishes and incubated at 37°C for 3–4 days. Similarly, 1 × 10^6^ RAW 264.7 cells were plated in 60 mm culture dishes and incubated at 37°C for 2–3 days. Both confluent cells were incubated with an ethanol-containing medium for 48 h to induce hepatic injury. Ethanol medium comprised of DMEM, 1% antibiotic-antimycotic solution, and 100 mM ethanol. Therefore, we assigned HepG2 cells and RAW 264.7 cells to 1 to 5 groups: (a) control group of untreated HepG2 cells and RAW 264.7 cells; (b) an ethanol control group of HepG2 cells and RAW 264.7 cells treated with 100 mM ethanol for 48 h to induce hepatic injury; (c) a test group1: HepG2 cells and RAW 264.7 cells were treated with 3 ml DMEM containing 1 mM Met concentration along with 100 mM ethanol for 48 h; (d) another test group2: HepG2 cells and RAW 264.7 cells were treated with 3 ml DMEM containing 10 *μ*l/ml probiotic V along with 100 mM ethanol for 48 h; (e) combination group: HepG2 cells and RAW 264.7 cells were treated with 10 *μ*l/ml probiotic V and 1 mM Met diluted in 3 ml DMEM along with 100 mM ethanol for 48 h. All the groups underwent Oil red O staining, oxidative stress estimation, ER stress quantification, ROS determination, and cytokine expression analysis.

### 2.5. In Vivo Chronic Ethanol Feeding with Concomitant Probiotic Visbiome® and Metformin Administration

Young male Wistar rats (8 to 10 weeks old) weighing 150-200 g were procured from Zydus-Cadila Pharmaceutical Industries Pvt. Ltd (India). Rats were lodged in standard cages (two rat/cage) and fed a normal chow diet before the initiation of the experiment with Lieber-DeCarli liquid diet feeding. All animals involving procedures were approved by the Committee for the Purpose of Control and Supervision of Experiments on Animals (CPCSEA) and Institutional Animal Ethics Committee (IAEC).

Weight and age-matched rats were given an ethanol-fed and pair-fed Lieber-DeCarli diet. They were acclimatized with Lieber-DeCarli's liquid diet for the first 2 days. Ethanol-fed groups were permitted free access to a complete Lieber-DeCarli liquid diet comprising ethanol. In this experiment, a pair-fed diet containing maltodextrin (substituted isocalorically) was given to the control rats for the entire feeding period. Ethanol-induced hepatic injury model (25 days, 32% total calories) contained increased concentrations of ethanol (vol/vol): 1% (2 days), 2% (2 days), 4% (7 days), 5% (7 days), and lastly 6% (7 days) [25].

The 6% (vol/vol) Lieber-DeCarli liquid diet containing ethanol provides 32% of total calories in the diet. Probiotic V, as well as Met, was provided to rats at doses of 10^8^ CFU/day and 75 mg/kg, respectively, by oral gavage over a specific period of ethanol feeding and pair-fed feeding. Following ethanol and pair-fed exposure feeding protocols, blood was taken from the posterior vena cava by syringe and ejected into tubes containing EDTA. Overnight-fasted rats were euthanized by exsanguination method (as mentioned in CPCSEA guidelines), and livers were excised. Other small portions of the liver were fixed in formalin, frozen in optimal cutting temperature (OCT) medium, and stored in RNA later at -20°C for isolating RNA. From whole blood, serum was isolated and kept at -80°C, until further use.

### 2.6. ROS Estimation

To estimate oxidative stress caused by ethanol in the presence or absence of probiotic V and Met, the control and treated cells are treated with ethanol and probiotic V and Met for 48 h. After treatment, cells were incubated with carboxy-H2-DCFDA in the dark with an absolute concentration of 30 *μ*M at 37°C for 1 hour. The cells were then scraped, and 200 *μ*l of the cell sample was added to a microtiter plate, and the fluorescence of the sample was measured at an excitation wavelength -485 nm and an emission wavelength -530 nm for approximating the total of ROS production using a fluorescence spectrophotometer (Perkin Elmer LS-55, USA) [26].

### 2.7. Oxidative Stress Estimation: MDA Analysis by HPLC Method

Control and treated cells were washed with 1 ml PBS to remove the culture medium. Then, the cells were centrifuged at 220 × g for 5 min at 4°C. The supernatant was removed, and the obtained pellet was resuspended in 200 *μ*l of PBS. Then, cells were sonicated for 7 min at RT, to lyse the cell membrane and release the total MDA, further centrifuged at 3500 × g for 15 min at 4°C. Samples containing sonicated cells (500 *μ*l) were incubated with 6 M sodium hydroxide (NaOH) (100 *μ*l) for 45 min in a water bath at 60°C. The samples were then acidified with 35% perchloric acid (250 *μ*l). The hydrolyzed samples were centrifuged at 15000 × g for 10 min. Then, supernatant (250 *μ*l) was collected and mixed with 2,4-dinitrophenylhydrazine (DNPH) (25 *μ*l) solution. It was further incubated in the dark for 10 mins. By HPLC (Waters Breeze-2, USA), the resulting samples were analyzed through the ODS2 reverse phase column by HPLC (Waters Breeze-2, USA). Acetonitrile and HPLC grade water having 0.2% acetic acid in the ratio 38 : 62, respectively, was used as a mobile phase. HPLC was done under isocratic conditions with a flow rate of 0.5 ml/min, and MDA in the sample was detected at 310 nm using a UV detector.

*Standard curve preparation*: 20 nmol/ml of MDA standard solution prepared from TEP (TCI, Japan) which was further diluted with 1% H_2_SO_4_ to yield a final concentration of 0.10, 0.20, 0.31, 0.62, 1.25, 2.50, 5.00, and 10.00 nM/ml of MDA. To 250 *μ*l of the standard, 25 *μ*l of DNPH was supplemented and incubated in the dark for 10 min. The samples were analyzed by HPLC using the same procedure as used for cell culture samples [27].

### 2.8. Determination of Lipid Accumulation through Oil Red O Staining in HepG2 Cells

To understand the changes occurring in control and treated cells due to the accumulation of lipids in the cells and to evaluate the reduction in ethanol-induced hepatic injury, Oil red O staining was performed. After 48 h of incubation, the control and treated cells were incubated for 1 hour at room temperature (RT) with 1 ml of 10% neutral buffered formalin. The cells were then rinsed twice with milli-Q. Oil red O stain (stock solution: 0.5% in 100 ml isopropanol and 1 ml; working solution: 3 : 2 ratio diluted in milli-Q) was added to the cells. The cells were incubated at RT for 15 mins. The cells were washed with milli-Q twice to eliminate the excessive stains. The cells were then counterstained with Cole's hematoxylin solution for 30-45 min. The additional stain was removed by washing the cells with milli-Q. Cells were observed and snapped under an inverted microscope (Magnus, India) using PBS as a mounting medium.

*Oil red O stain extraction by isopropanol method:* to determine the difference in the lipid accumulation between control and treated cells, after staining the cells with Oil red O, the stain was extracted with absolute isopropanol. The extracted Oil red O stain was measured spectrophotometrically at 570 nm using isopropanol as blank [28].

### 2.9. Measurement of Serum Liver Damage Indices and Lipid Components

Serum samples were measured for alanine aminotransferase (ALT), aspartate aminotransferase (AST), and alkaline phosphatase (ALP). To check the level of alcohol-induced abnormality in the body's lipid metabolism, the serum levels of total cholesterol and triglyceride were measured using enzymatic kits available commercially and followed according to the kit manufacturer's protocol. All the experiments were performed in triplicate, and the respective concentrations were determined.

### 2.10. Lipid Analysis

Lipids present in the liver were extracted by using a ratio of 2 : 1 v/v chloroform : methanol solution mixed to a fixed amount of liver tissue and homogenized properly. The homogenate was centrifuged at 5000 rpm for 10 min. The lipid-containing bottom solutions were collected, and then, pellets were obtained by evaporating chloroform via centrifugation for 2 h using a centrifugal vacuum concentrator. Later, lipids were solubilized using chloroform, and then, hepatic triglyceride and total cholesterol were measured using a quantitative measurement kit and followed according to the kit manufacturer's protocol [29].

### 2.11. Isolation of RNA, Synthesis of cDNA, and qRT-PCR

Total RNA was isolated from the control and treated cells as well as liver using TRIzol reagent. The purity and concentration of RNA in the sample were measured at 260 nm, and the A260/A280 ratio of the sample was determined using Nanodrop 2000 (Thermofisher Scientific, USA). DNA contamination in the RNA sample was removed by treating the RNA sample with DNase I enzyme. From complete RNA, around 1 *μ*g was used to synthesize cDNA using a first-strand cDNA synthesis kit. For qPCR, the maxima SYBR Green/ROX qPCR master mix was used to quantify the mRNA expression levels for all genes under analysis in the Agilent Strategene Mx3005P system. The qRT-PCR system was containing 1 *μ*l cDNA, 0.5 *μ*l of 10 *μ*M FP (forward primers), 0.5 *μ*l of 10 *μ*M RP (reverse primers), 10 *μ*l SYBR green master mix, and 8.0 *μ*l milli-Q water making a 20 *μ*l reaction system. The 2^-*ΔΔ*Ct^ method (fold change over basal) was applied to evaluate mRNA expression levels in HepG2-treated cells and liver tissue [30]. 18S rRNA was presented as an internal reference gene control. The lists of primer sequences for different sets used are tabulated in Tables 1–3.

### 2.12. Histopathological Observation of the Liver

The formalin-fixed liver section was processed in optimal cutting temperature (OCT) medium. 5-10 *μ*M sections were cut using a cryostat. The paraffin sections were stained with hematoxylin-eosin (HE) solution to recognize the changes in hepatocyte morphology among the rats treated differently. All sections of slides were examined under an optical microscope by a single investigator who was blinded to the treatment status.

### 2.13. Determination of Protein Levels of Nrf-2 and HO-1 in an In Vitro and In Vivo Model of Ethanol-Induced Hepatic Injury

- In HepG2 cells, cell-free supernatants were used to analyze Nrf-2 and HO-1 levels. After the treatment, from each test plate, cell culture media was aspirated out and centrifuged at 1500 rpm for 10 min at 4°C. The collected supernatant was analyzed according to the manufacturer's instructions (Abcam plc, UK) and measured at 450 nm. Nrf-2 and HO-1 protein levels were measured at picograms per milliliter
- To analyze Nrf-2 levels in the ethanol-induced hepatic injury model, the nuclear extract was isolated from the rat liver tissue. Liver homogenates were incubated in the lysis buffer [10 mM HEPES; pH 7.5, 10 mM KCl, 0.1 mM EDTA, 1 mM dithiothreitol (DTT), 0.5% Nonidet-40, and 0.5 mM PMSF along with the protease inhibitor cocktail] and allowed to swell on ice for 20 min with sporadic mixing. Samples were vortexed and centrifuged at 10,000 × g at 4°C for 10 min. The supernatant obtained was used as a cytoplasmic extract. Later, the pellet was washed with the lysis buffer and resuspended in the nuclear extraction buffer [20 mM HEPES (pH 7.5), 400 mM NaCl, 1 mM EDTA, 1 mM DTT, and 1 mM PMSF with protease inhibitor cocktail] and incubated in ice for 30 min. Nuclear extract was collected by centrifugation at 12,000 × g for 15 min at 4°C. Proteins levels were checked using Bradford reagent [31]

To analyze HO-1 levels in the ethanol-induced hepatic injury model, 100 mg of liver tissue was mixed with 1 ml PBS and homogenized. The homogenate was centrifuged at 5000 rpm for 10 min at 4°C, and the resultant supernatant was used as the liver tissue homogenate. Proteins levels were checked using Bradford reagent.

Collected cell-free supernatant, nuclear extract, and liver tissue homogenate were measured through enzyme-linked immunosorbent assay (ELISA) according to the protocol given by the kit manufacturer (Abcam plc, UK).

### 2.14. Statistical Analysis

All respective experiments were performed at least thrice. Data of the replicates were calculated as mean ± SD. The GraphPad Prism 7 software (GraphPad Software Inc., California Corporation) was used to analyze the results. The differences between all groups were analyzed using a one-way analysis of variance (ANOVA). Variations between groups were considered to be significant at *p* values less than 0.05.

## 3. Results

### 3.1. Combination of Probiotic V and Met Improves the Viability of HepG2 Cell Line

Toxicity of probiotic V (10, 50, and 100 *μ*l/ml) and Met (1, 2, and 3 mM) individually on HepG2 cell was assessed in the presence and absence of 100 mM ethanol (Table 4). The effect of the combinatorial doses of probiotic V and Met (10 *μ*l/ml probiotic V with 1, 2, and 3 mM Met; 50 *μ*l/ml probiotic V with 1, 2, and 3 mM Met; and 100 *μ*l/ml probiotic V with 1, 2, and 3 mM Met) also was assessed as a measure of cell viability on HepG2 cells treated in the presence and absence of 100 mM ethanol (Figures 1(a) and 1(b)).

Neither the individual treatment with probiotic V nor with Met at different concentrations showed significant cell death of HepG2 cells in the absence of 100 mM ethanol. However, there was a slight reduction observed in the viability of the cells treated with 3 mM Met alone. Treatment with 100 mM ethanol on HepG2 cells induced cell death, and therefore, only 64.6% cell viability was observed after 48 h of 100 mM ethanol treatment as compared to the untreated cells (i.e., 95.7%). To see if probiotic V could prevent the ethanol-induced toxicity, HepG2 cells were treated with probiotic V in the presence of 100 mM ethanol. Our results indicate that probiotic V treatment at different concentrations (10, 50, and 100 *μ*l/ml) shows a dose-dependent increase in cell viability (81.9%, 83.1%, and 85.5%, respectively) when compared to ethanol-exposed HepG2 cells at 48 h. The effect of Met at various concentrations in the presence of 100 mM ethanol also was assessed as a measure of cell viability of HepG2 cells. The viability of cells exposed to 100 mM ethanol in the presence of Met alone showed a dose-dependent decrease in cell viability (82.7%, 81.6%, and 67.9%, respectively) when compared to ethanol-exposed HepG2 cells at 48 h, which is suggestive of the toxicity of Met at higher concentration.

As the treatment with probiotic V alone showed a dose-dependent increase, and the treatment with Met alone showed a dose-dependent decrease in the viability, we checked if the combination of probiotic V and Met could work in synergy to improve the cell viability of HepG2 when treated in the presence of 100 mM ethanol. The combinatorial doses of probiotic V and Met did not significantly affect the viability of HepG2 cells treated in the absence of 100 mM ethanol across all the different combinations. However, we observed that probiotic V treatment at various concentrations rescued the toxicity of 3 mM Met and improved the cell viability. This suggests that probiotic V can improve the efficacy of metformin by reducing its toxicity at a higher concentration. Also, the viability of the cells treated with 100 mM ethanol in the presence of different combinations of probiotic V and Met was improved as compared to ethanol-treated HepG2 cells in the absence of probiotic V and Met, which suggests the beneficial role of probiotic V and Met as combinatorial treatment. The most significant difference in the cell viability was observed with a combination of 100 *μ*l/ml probiotic V and 1 mM Met (87.3%), but this difference was not significantly different when compared to the cells treated with only 100 *μ*l/ml probiotic V in the presence of ethanol (85.5%). As our study is aimed at seeing if probiotic V and Met could work in synergy to prevent the ethanol-induced toxicity of HepG2 cells, we chose the combination of 10 *μ*l/ml probiotic V and 1 mM Met, because this combination significantly improved the cell viability of ethanol-exposed HepG2 cells, compared to their respective individual treatments.

### 3.2. Combination of Probiotic V and Met Improves the Morphology in HepG2 Cell Line

**Figure 2:**
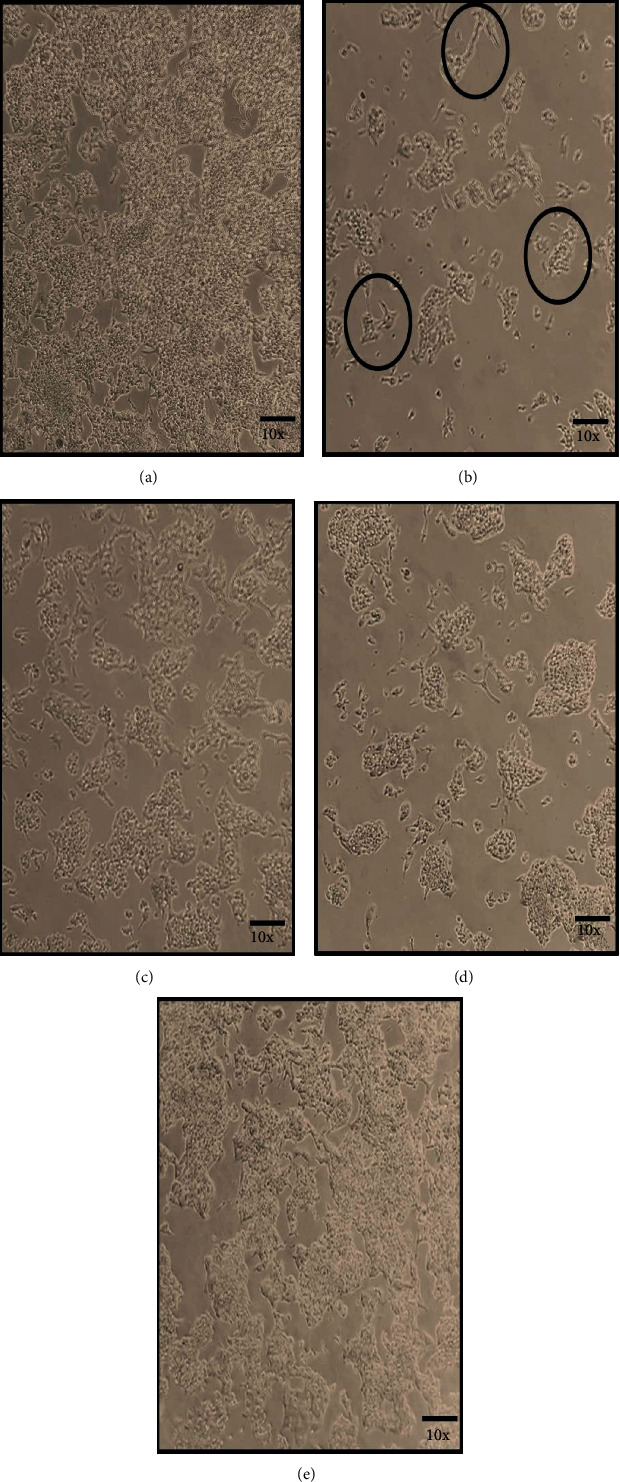
HepG2 cells were treated with probiotic V and Met alone or together as probiotic V and Met treatment in the presence of ethanol for 48 h. Microscopic images of HepG2 cells treated with (a) control, (b) 100 mM ethanol control, (c) 100 mM ethanol+10 *μ*l/ml probiotic V, (d) 100 mM ethanol+1 mM Met, and (e) 100 mM ethanol+10 *μ*l/ml probiotic V and 1 mM Met combination. The above figures are illustrative 10x objective images of three individual experiments. The control cells were well adhered to the surface of the cultured cells and displayed the normal epithelial morphology of HepG2 cells. In contrast, the majority of HepG2 cells treated with ethanol changed their normal shape and became round and shrunken and could not remain affixed to the walls of the culture plate. Co-administration of probiotic V and Met was more effective in preventing these morphological changes in HepG2 cells as compared to the individual treatment of either probiotic V and Met.

The HepG2 cells when treated with ethanol showed a change in their morphology as seen by the constricted cell membrane and distorted shape of the cells (Figure 2(b)) as compared to the epithelial shape of the control cells. Met or probiotic V-treated cells in absence of ethanol also maintained the normal epithelial shape similar to that of untreated control HepG2 cells. Administration of probiotic V and Met alone prevented the cellular injury caused due to ethanol by maintaining their original shape. However, probiotic V and Met, in combination (Figure 2(d)), ameliorated the ethanol-induced damage comparatively better than the individual treatment of probiotic V or Met.

### 3.3. Combination of Probiotic V and Met Ameliorates the Liver Histopathological Changes in an In Vivo Model of Ethanol-Induced Hepatic Injury

**Figure 3:**
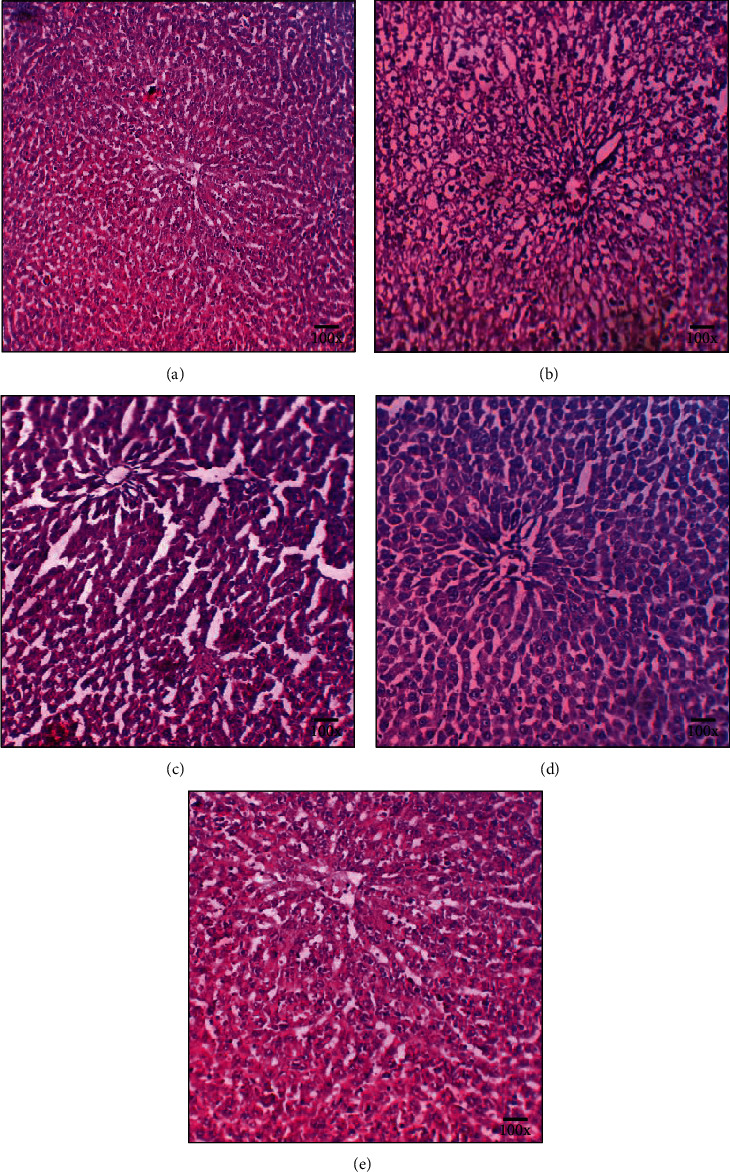
Probiotic V and Met alone or in combination regulates the lipid accumulation in the liver to prevent ethanol-induced hepatic injury. Histological examination of liver sections stained with hematoxylin and eosin (100x magnification) of (a) control, (b) ethanol-fed, (c) Met (75 mg/kg)+ethanol-fed, (d) probiotic V (10^8^ CFU/day)+ethanol-fed, and (e) probiotic V (10^8^ CFU/day)+Met (75 mg/kg)+ethanol-fed rats.

Histological analysis of liver tissue exhibited that the control rats depict the normal histology (hepatic cells with clear cytoplasm, nucleus, nucleolus, and central vein). The ethanol-fed rats demonstrated a dramatic increase in fat droplets with cellular inflammatory infiltrations and disorderly arrangements of hepatocytes as the representative pathological change compared to the control group. Compared with this, Met (75 mg/kg) depicted congested central vein and disorderly arranged hepatocytes. On the other hand, probiotic V (10^8^ CFU/day) demonstrated normal hepatocytes and central vein with full restoration. Moreover, feeding probiotic V and Met in combination could significantly prevent hepatic lipid accumulation and safeguard the hepatic cells as compared to probiotic V or Met alone (Figure 3). These results depicted that combined treatment of probiotic V and Met help in restoring the normal hepatic architecture.

### 3.4. Combination of Probiotic V and Met Prevents Oxidative Stress in HepG2 Cell Line

**Figure 4:**
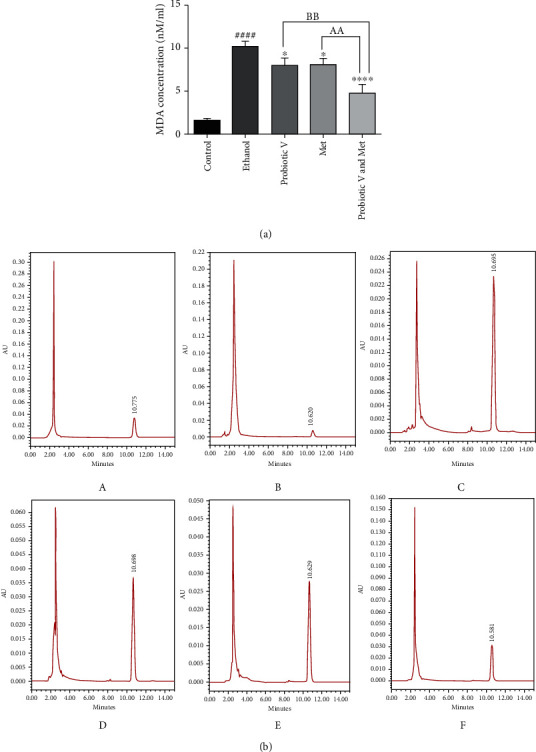
(a) MDA estimation in HepG2 cells by HPLC method. Here, 100 mM ethanol-exposed HepG2 cells were treated with 100 *μ*l/ml probiotic V and 1 mM Met alone or together as probiotic V and Met combination for 48 h. The amount of MDA was quantified with reference to the standard area under the curve (AUC), and values represent the mean ± SD of three individual experiments. Statistical significance was assessed by one-way ANOVA followed by Tukey *post hoc* test. Statistical analysis: ^####^*p* < 0.0001 compared with control group; ^∗^*p* < 0.05 and ^∗∗∗∗^*p* < 0.0001 compared with ethanol group; ^AA,BB^*p* < 0.01 compared with probiotic V and Met combinatorial group; (b) HPLC chromatograms of cells MDA after DNPH derivatization are shown as follows (A) MDA standard, (B) control, (C) 100 mM ethanol, (D) 100 mM ethanol+1 mM Met, (E) 100 mM ethanol+10 *μ*l/ml probiotic V, and (F) 100 mM ethanol+10 *μ*l/ml probiotic V and 1 mM Met combination. HepG2 cells were treated with ethanol alone or along with probiotic V and Met alone or together as probiotic V and Met combination for 48 h. After 48 h of incubation, hydrolyzed cells were mixed with DNPH and analyzed by HPLC using the ODS2 reverse-phase column. Acetonitrile and autoclaved milli-Q water containing 0.2% acetic acid in the ratio 38 : 62, respectively, was used as mobile phase.

The concentration of MDA levels using the HPLC method in ethanol-exposed HepG2 cells was found to be increased (10.17 ± 0.38 nM/ml) when compared with control cells (1.54 ± 0.28 nM/ml). Co-administration with probiotic V and Met in combination reduced the levels of MDA more efficiently (4.76 ± 0.8 nM/ml) as compared to ethanol-exposed HepG2 cells. However, there was a significant difference in the levels of MDA in either probiotic V (8.04 ± 0.53 nM/ml) or Met (8.04 ± 0.55 nM/ml) treated groups as compared to the combinatorial dose of probiotic V and Met in ethanol-exposed HepG2 cells (Figure 4). Therefore, present data reveal that probiotic V and Met in combination are beneficial in preventing ethanol-induced oxidative stress.

### 3.5. Combination of Probiotic V and Met Inhibits ROS Production in HepG2 Cell Line

**Figure 5:**
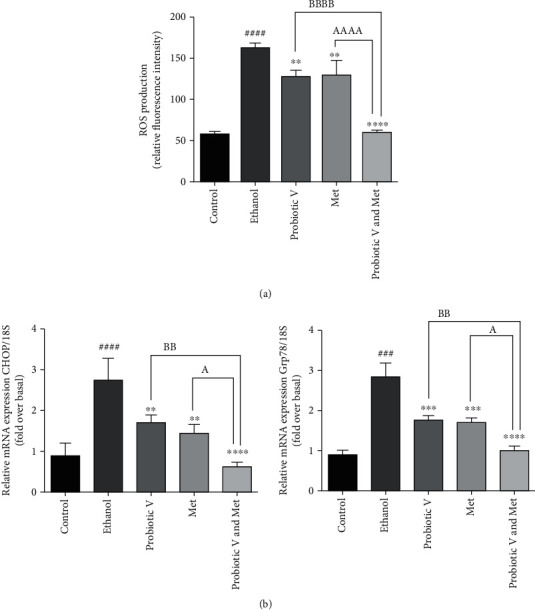
Effect of probiotic V and Met alone or together as probiotic V and Met combination on ROS production and ER stress upon the exposure to ethanol-exposed HepG2 cells by (a) fluorescence spectroscopy with excitation/emission at 495 nm/529 nm after incubation with carboxy-H_2_-DCFDA and (b) mRNA expression of ER stress genes (CHOP and Grp78) in HepG2 cells. The gene expression levels were calculated after stabilizing against 18S rRNA in every sample and are presented as relative mRNA expression units. Values represent the mean ± SD of three individual experiments. Statistical significance was assessed by one-way ANOVA followed by Tukey *post hoc* test. Statistical analysis: ^###^*p* < 0.001 and ^####^*p* < 0.0001 compared with control group; ^∗∗^*p* < 0.01, ^∗∗∗^*p* < 0.001, and ^∗∗∗∗^*p* < 0.0001 compared with ethanol group; ^A^*p* < 0.05, ^BB^*p* < 0.01, and ^AAAA,BBBB^*p* < 0.0001 compared with probiotic V and Met combinatorial group.

ROS production was evaluated through fluorescence spectroscopy in HepG2 cells exposed to ethanol alone and ethanol in the presence of probiotic V and Met individually, as well as in combination, and was then incubated with carboxy-H2-DCFDA at the final concentration of 30 *μ*M at 37°C for 1 hour in the dark. Cotreatment of ethanol-exposed cells with probiotic V and Met in combination was associated with reduced ROS production in comparison with HepG2 cells exposed to ethanol alone, as well as compared to the individual treatment of probiotic V or Met (Figure 5(a)). Therefore, present data reveal that probiotic V and Met in combination are beneficial in ameliorating ethanol-induced ROS generation.

### 3.6. Combination of Probiotic V and Met Suppresses Ethanol-Induced ER Stress in HepG2 Cell Line

Chronic alcohol consumption leads to ER stress resulting in the activation of unfolded protein response (UPR) [22]. Expression of mRNA levels of ER stress markers like glucose regulatory protein 78 (Grp78) and CCAAT enhancer-binding protein homologous protein (CHOP) revealed that ethanol-exposed HepG2 cells showed increased ER stress in 48 h. In the presence of 100 mM ethanol, administration of probiotic V and Met alone or in combination showed reduced expression levels of ER stress genes as compared to ethanol-exposed HepG2 cells. Cotreatment of probiotic V and Met showed a significant difference in ER stress genes when compared with the individual treatment of either probiotic V or Met (Figure 5(b)). Therefore, combinatorial treatment of probiotic V and Met attenuates the ethanol-induced ER stress.

### 3.7. Combination of Probiotic V and Met Reduces Ethanol-Induced Lipid Accumulation in HepG2 Cell Line

**Figure 6:**
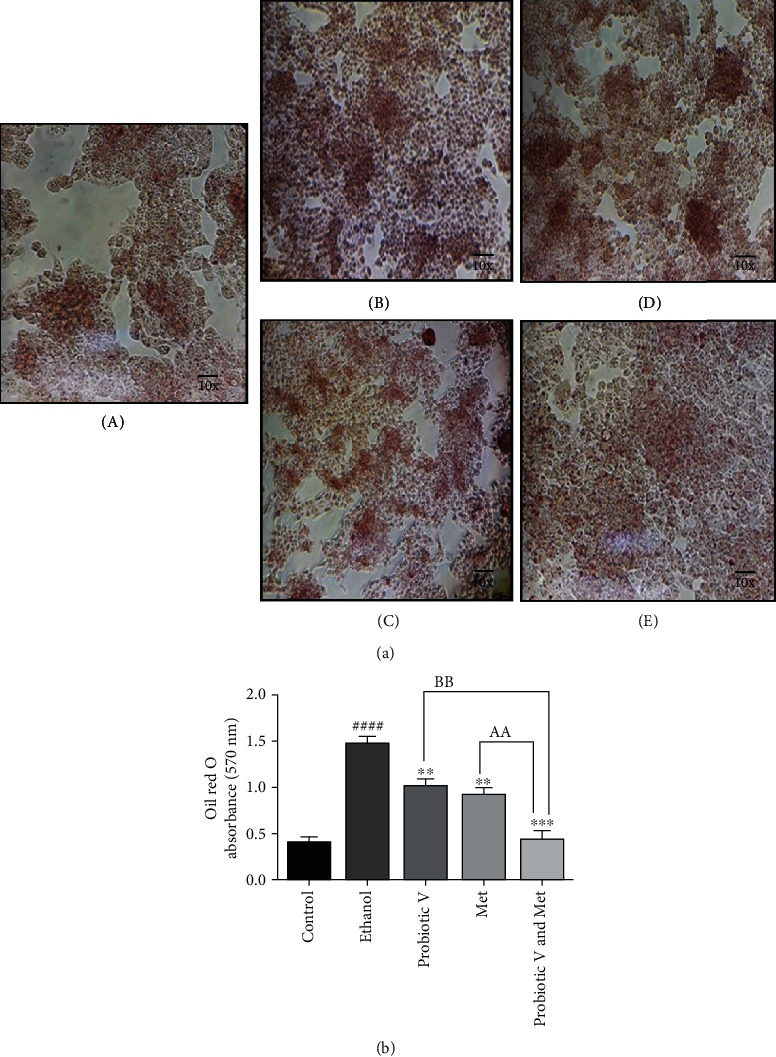
(a) Microscopic images of Oil red O staining showing the results of (A) control, (B) 100 mM ethanol control, (C) 100 mM ethanol+10 *μ*l/ml probiotic V, (D) 100 mM ethanol+1 mM Met, and (e) 100 mM ethanol+10 *μ*l/ml probiotic V and 1 mM Met combination exposed HepG2 cells showed increased lipid accumulation. The above images are representative of three different experiments at the 10x objective. (b) Spectrophotometric analysis results of lipid accumulation in HepG2 cells by Oil red O extraction method. Ethanol-exposed HepG2 cells cotreated with probiotic V and Met showed decrease lipid accumulation when compared to ethanol-exposed cells. Results are mean ± SD (*n* = 3 individual experiments). Statistical significance was assessed by one-way ANOVA followed by Tukey *post hoc* test. Statistical analysis: ^####^*p* < 0.0001 compared with control group; ^∗∗^*p* < 0.01 and ^∗∗∗^*p* < 0.001 compared with ethanol group; ^AA,BB^*p* < 0.01 compared with probiotic V and Met combinatorial group.

Accumulation of lipids was analyzed through Oil red O staining of cells microscopically and quantified using a spectrophotometer at 570 nm by isopropanol extraction method (Figure 6(a)). To determine whether probiotic V and Met alone or in combination can help in reducing the amount of accumulated lipids in HepG2 cells, probiotic V and Met were exposed individually and in combination along with 100 mM ethanol for 48 h. The total accumulation of lipids in the cells after the exposure of probiotic V and Met alone or in combination along with ethanol was measured by Oil red O extraction process.

The total lipid accumulation was found to be elevated in ethanol-exposed HepG2 cells compared to control cells. Upon administration of probiotic V and Met alone as well as in combination with ethanol-exposed HepG2 cells, the amount of lipid accumulated was decreased at 48 h. The results demonstrated that complete lipid accumulation was observed in ethanol-exposed HepG2 cells, which was restored by probiotic V and Met in combination, with better efficacy as compared to the individual treatment of either probiotic V or Met (Figure 6(b)).

### 3.8. Combination of Probiotic V and Met Ameliorates the Ethanol-Induced Upregulation of Liver Enzyme Levels in Serum and Lipid Profile Markers in an In Vivo Model of Hepatic Injury

ALT, AST, and ALP are fundamental biochemical markers indicative of liver injury. The present study showed a significant increase in serum transaminases (ALT and AST) and ALP levels in the ethanol-fed rats compared to the control group. The elevated serum levels can be due to the outflow of cellular enzymes into the bloodstream. Treatment with either probiotic V or Met significantly reduced the serum levels of ALT, AST, and ALP as compared to the ethanol-fed group, which was further downregulated by the two in the combination when compared with the ethanol-fed group as well as individual treatment of probiotic V or Met.

**Figure 7:**
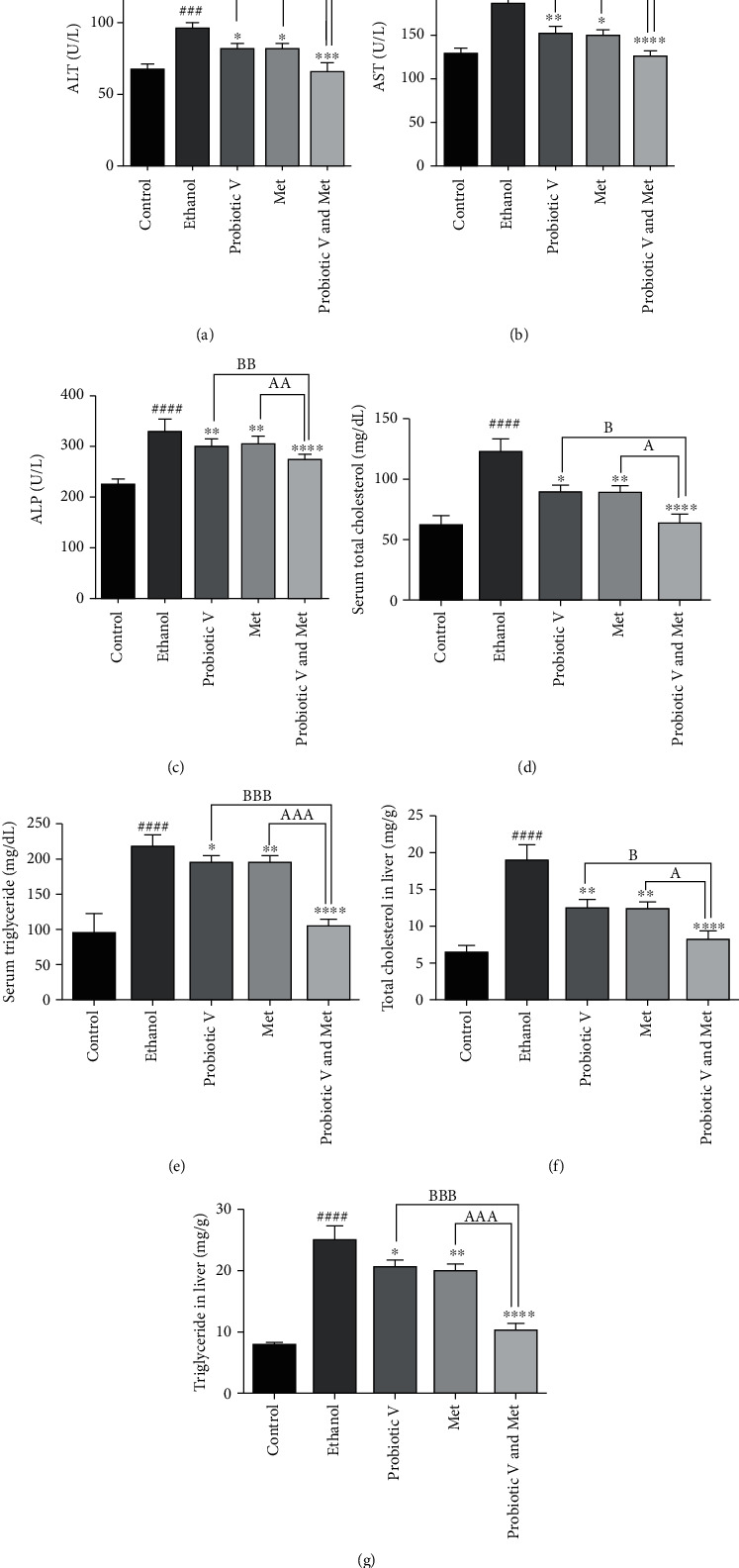
Effect of probiotic V and Met alone or in combination on ethanol-induced liver injury. Serum levels of liver enzymes, i.e., (a) ALT, (b) AST, (c) ALP, and lipid markers, i.e., (d) serum cholesterol, (e) serum triglycerides, (f) hepatic cholesterol, and (g) hepatic triglycerides, were determined using a microplate reader. Values are expressed as mean ± SD of six rats. Statistical significance was assessed by one-way ANOVA followed by Tukey *post hoc* test. Statistical analysis: ^###^*p* < 0.001 and ^####^*p* <0.0001 compared with control group; ^∗^*p* < 0.05, ^∗∗^*p* < 0.01, and ^∗∗∗∗^*p* < 0.0001 compared with ethanol group; ^A,B^*p* < 0.05, ^AA,BB^*p* < 0.01, and ^AAA,BBB^*p* < 0.001 compared with probiotic V and Met combinatorial group. ALT: alanine aminotransferase; AST: aspartate aminotransferase; ALP: alkaline phosphatase.

Also, serum levels, as well as hepatic contents of total cholesterol (TC) and triglyceride (TG) in the ethanol-fed rats, were significantly higher compared to those in the control group, which were dramatically reduced in the combinatorial treatment of probiotic V and Met in comparison to the ethanol-fed group as well as individual treatment of probiotic V or Met (Figure 7). These results indicate that the combined treatment of probiotic V and Met could improve liver metabolic function in ethanol-fed rats.

### 3.9. Combination of Probiotic V and Met Regulates Lipid Metabolism in an In Vitro and an In Vivo Model of Ethanol-Induced Hepatic Injury

Chronic alcohol consumption leads to abnormal lipid metabolism in the liver by decreased adenosine monophosphate-activated protein kinase (AMPK) activation which leads to increased sterol regulatory element binding protein 1c (SREBP-1c) expression, further leading to increased lipogenesis through activation of downstream lipogenic genes like acetyl-CoA carboxylase (ACC) and fatty acid synthase (FAS) in ethanol-exposed HepG2 cells as well as ethanol-fed rats. Individual treatment of either probiotic V or Met administration activates adenosine monophosphate-activated protein kinase (AMPK), a critical regulator of lipid metabolism, which is otherwise inhibited in the presence of ethanol-exposed HepG2 cells and ethanol-fed rats. Activation of AMPK by either treatment of Met or probiotic V inhibits the expression of transcription factor, i.e., sterol regulatory element binding protein 1c (SREBP-1c) (also a key regulator of lipid metabolism), thereby inhibiting the ethanol-induced lipogenesis. Consistent with the altered expression of SREBP-1c, it also decreases the expression of downstream lipogenic enzymes like acetyl-CoA carboxylase (ACC) and fatty acid synthase (FAS) as compared to ethanol-exposed HepG2 cells as well as ethanol-fed rats, which was more significantly reduced as compared to either individual treatment of probiotic V or Met.

**Figure 8:**
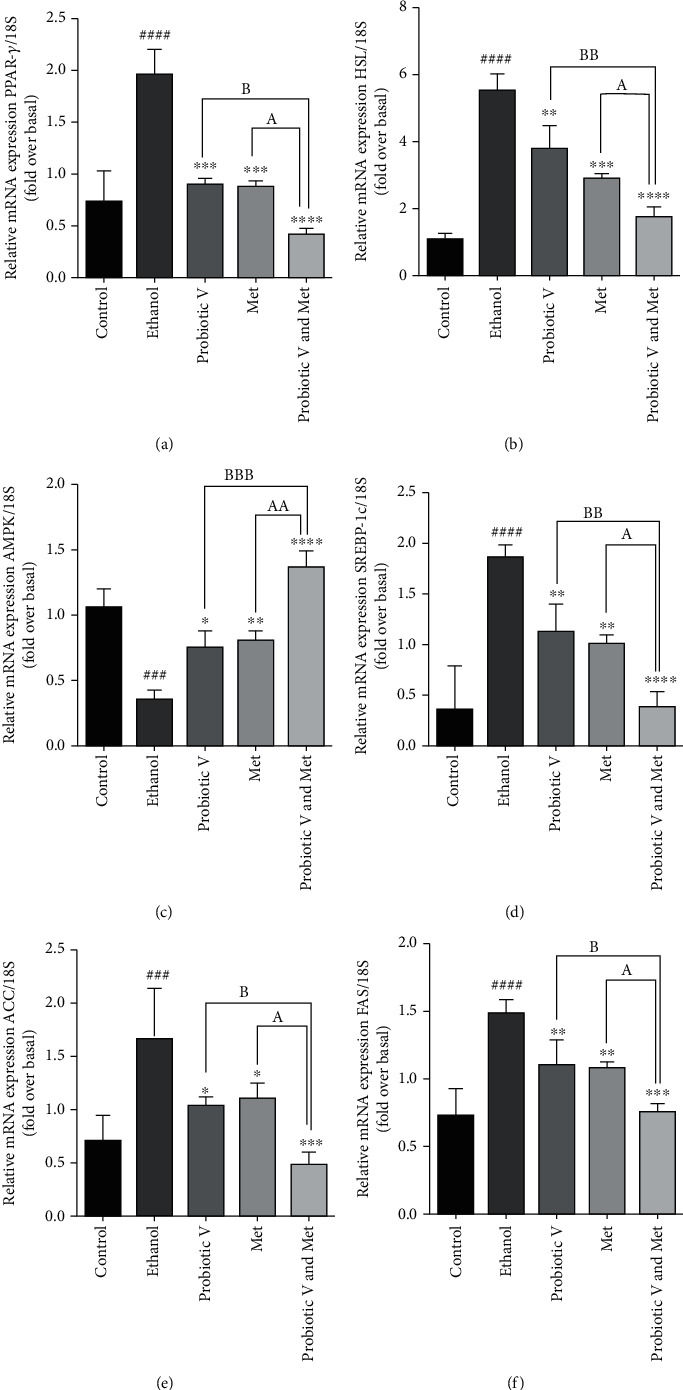
qRT-PCR analysis of (a) PPAR-*γ*, (b) HSL, (c) AMPK, (d) SREBP-1c, (e) ACC, and (f) FAS genes from ethanol-exposed HepG2 cells along with probiotic V and Met alone or together as probiotic V and Met combination for 48 h. The gene expression levels were calculated after stabilizing against 18S rRNA in every sample and are represented as relative mRNA expression units. Values represent the mean ± SD of three individual experiments. Statistical significance was assessed by one-way ANOVA followed by Tukey post hoc test. Statistical analysis: ^###^*p* < 0.001 and ^####^*p* < 0.0001 compared with control group; ^∗^*p* < 0.05, ^∗∗^*p* < 0.01, ^∗∗∗^*p* < 0.001, and ^∗∗∗∗^*p* < 0.0001 compared with ethanol group; ^A,B^*p* < 0.05, ^AA,BB^*p* < 0.01, and ^AAA,BBB^*p* < 0.001 compared with probiotic V and Met combinatorial group. PPAR-*γ*: proliferator-activated receptor-gamma; HSL: hormone sensitive lipase; AMPK: adenosine monophosphate-activated protein kinase; SREBP-1c: sterol regulatory element binding protein 1c; ACC: acetyl-CoA carboxylase; FAS: fatty acid synthase.

**Figure 9:**
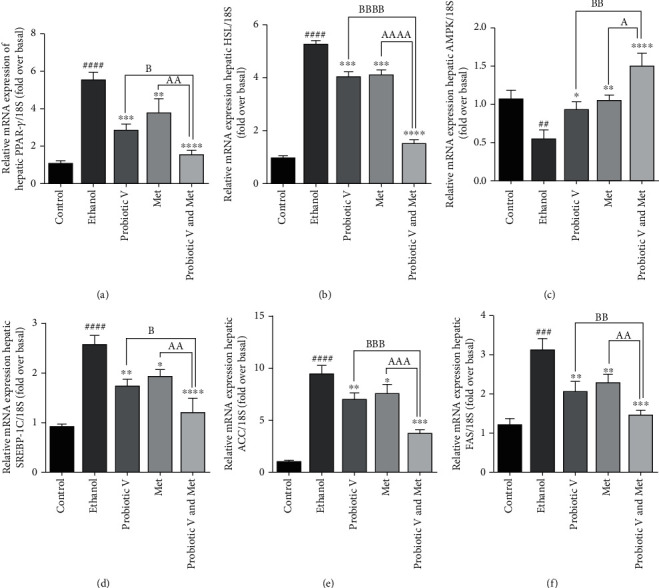
Effect of probiotic V and Met alone or in combination regulates the lipid metabolism in ethanol-induced hepatic injury. Hepatic levels of lipogenic markers, i.e., (a) PPAR-*γ*, (b) HSL, (c) AMPK, (d) SREBP-1c, (e) ACC, and (f) FAS. Values are expressed as mean ± SD of six rats. Statistical significance was assessed by one-way ANOVA followed by Tukey *post hoc* test. Statistical analysis: ^##^*p* < 0.01, ^###^*p* < 0.001, and ^####^*p* < 0.0001 compared with control group; ^∗^*p* < 0.05, ^∗∗^*p* < 0.01, ^∗∗∗^*p* < 0.0001, and ^∗∗∗∗^*p* < 0.0001 compared with ethanol group; ^A,B^*p* < 0.05, ^AA,BB^*p* < 0.01, ^AAA,BBB^*p* < 0.001, and ^AAAA,BBBB^*p* < 0.0001 compared with probiotic V and Met combinatorial group. PPAR-*γ*: proliferator-activated receptor-gamma; HSL: hormone sensitive lipase; AMPK: adenosine monophosphate-activated protein kinase; SREBP-1c: sterol regulatory element binding protein 1c; ACC: acetyl-CoA carboxylase; FAS: fatty acid synthase.

In the liver, PPAR-*γ* and HSL are present at basal levels; however, treatment with ethanol upregulates the hepatic expression of PPAR-*γ* and HSL in comparison to the control group, leading to increased lipogenesis. The treatment with either probiotic V or Met downregulated the expression levels of PPAR-*γ* and HSL which resulted in reduced lipogenesis as compared to the ethanol-exposed HepG2 cells as well as ethanol-fed rats. This was further diminished more significantly by the two in combination when compared with the ethanol group as well as probiotic V- and Met-unaided groups (Figures 8 and 9). Our study demonstrated that combinatorial treatment of probiotic V and Met in the presence of ethanol is effective in regulating lipid metabolism, thereby preventing ethanol-induced lipogenesis.

### 3.10. Combination of Probiotic V and Met Alleviates the Proinflammatory/Anti-Inflammatory/Oxidative Stress Markers in an In Vitro and In Vivo Ethanol-Induced Hepatic Injury

**Figure 10:**
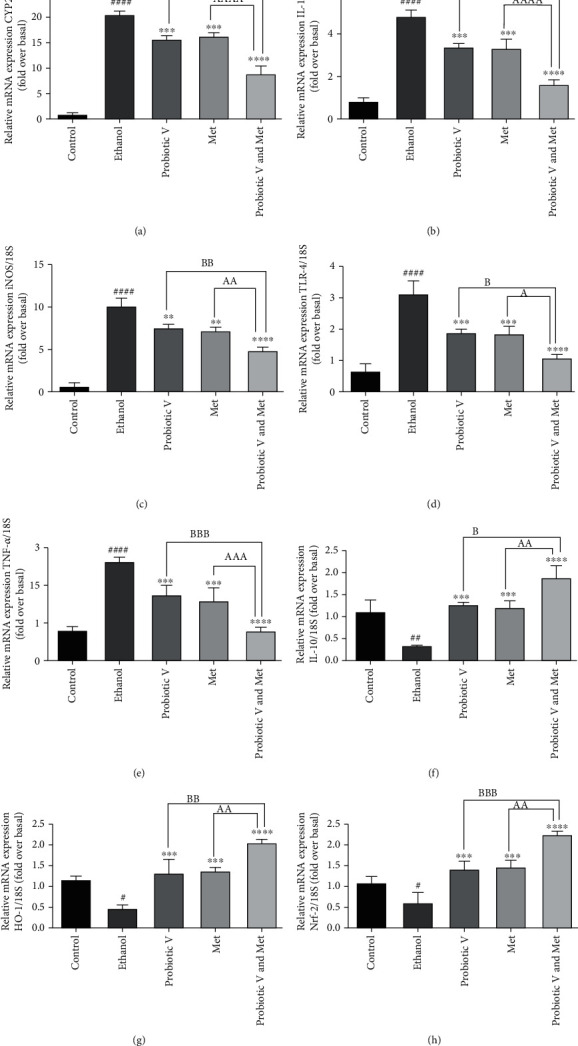
qRT-PCR analysis results of (a) CYP2E1, (b) IL-1*β*, (c) iNOS, (d) TLR-4, (e) TNF-*α*, (f) IL-10, (g) HO-1, and (h) Nrf-2 genes in ethanol-exposed HepG2 cells alone and along with probiotic V and Met alone or in combination treatment for 48 h. The gene expression levels were calculated after stabilizing against 18S rRNA in every sample and are presented as relative mRNA expression units. Values represent the mean ± SD of three individual experiments. Statistical significance was assessed by one-way ANOVA followed by Tukey *post hoc* test. Statistical analysis: ^#^*p* < 0.05, ^##^*p* < 0.01, and ^####^*p* < 0.0001 compared with control group; ^∗^*p* < 0.05, ^∗∗^*p* < 0.01, ^∗∗∗^*p* < 0.001, and ^∗∗∗∗^*p* < 0.0001 compared with ethanol group; ^A,B^*p* < 0.05, ^AA,BB^*p* < 0.01, ^AAA,BBB^*p* < 0.001, and ^AAAA,BBBB^*p* < 0.0001 compared with probiotic V and Met combinatorial group.

**Figure 11:**
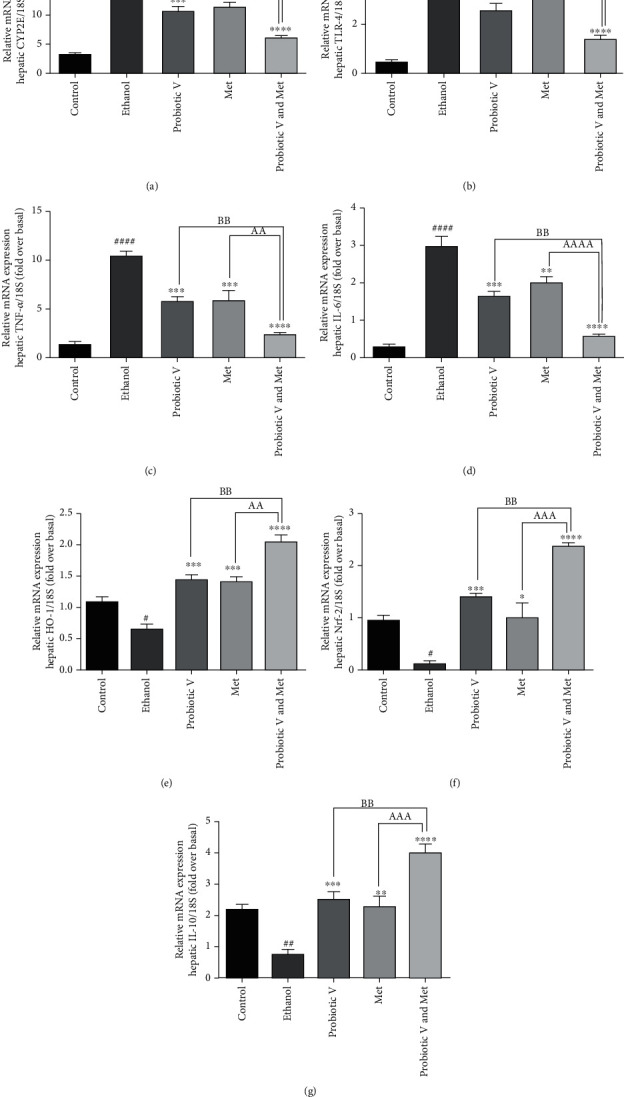
Effect of probiotic V or Met alone or in combination on ethanol-induced inflammatory response and oxidative stress. The inflammatory gene levels in the male Wistar rat liver are (a) CYP2E1, (b) TLR-4, (c) TNF-*α*, (d) IL-6, (e) HO-1, (f) Nrf-2, and (g) IL-10. Ethanol-fed rats were also treated with probiotic V (10^8^ CFU/day) and Met (75 mg/kg) alone or together in combination for 25 days. At the end of the feeding protocol, liver tissue was collected, RNA was isolated, and qRT-PCR was performed to quantify the relative mRNA expression levels of the genes. Values are expressed as mean ± SD of six rats. Statistical significance was assessed by one-way ANOVA followed by Tukey post hoc test. Statistical analysis: ^#^*p* < 0.05 and ^####^*p* < 0.0001 compared with control group; ^∗^*p* < 0.05, ^∗∗^*p* < 0.01, ^∗∗∗^*p* < 0.0001, and ^∗∗∗∗^*p* < 0.0001 compared with ethanol group; ^A,B^*p* < 0.05, ^AA,BB^*p* < 0.01, ^AAA,BBB^*p* < 0.001, and ^AAAA,BBBB^*p* < 0.0001 compared with probiotic V and Met combinatorial group.

Ethanol-induced hepatic injury can also cause chronic inflammation. Thus, we evaluated the effects of probiotic V and Met alone, as well as in combination on inflammatory response and oxidative stress markers (Figures 10 and 11). The mRNA expression level of an ethanol-metabolizing enzyme-like cytochrome P450 2E1 (CYP2E1), pro-inflammatory markers like IL-1*β*, TNF-*α*, inducible nitric oxide synthase (iNOS), and receptor mediating inflammatory response, i.e., TLR-4, was found to be upregulated, and mRNA expression levels of anti-inflammatory cytokine like IL-10 and oxidative stress markers like heme oxygenase-1 (HO-1) and nuclear factor erythroid 2-related factor 2 (Nrf-2) were found to be downregulated in ethanol-exposed HepG2 cells and ethanol-fed rats. We observed that mRNA expression levels of CYP2E1 and TLR-4 as wells pro-inflammatory markers were dramatically diminished, and anti-inflammatory cytokine IL-10 and oxidative stress markers (HO-1 and Nrf-2) were largely upregulated in ethanol-induced HepG2 cells and ethanol-fed rats treated with probiotic V and Met alone. However, probiotic V or Met in combination could additionally diminish the expression of pro-inflammatory markers and more significantly elevated the IL-10, HO-1, and Nrf-2 expression levels as compared to probiotic V or Met-unaided groups.

### 3.11. Combination of Probiotic V and Met Upregulates the Production of Nrf-2 and HO-1 Protein Levels in an In Vitro and In Vivo Ethanol-Induced Hepatic Injury

**Figure 12:**
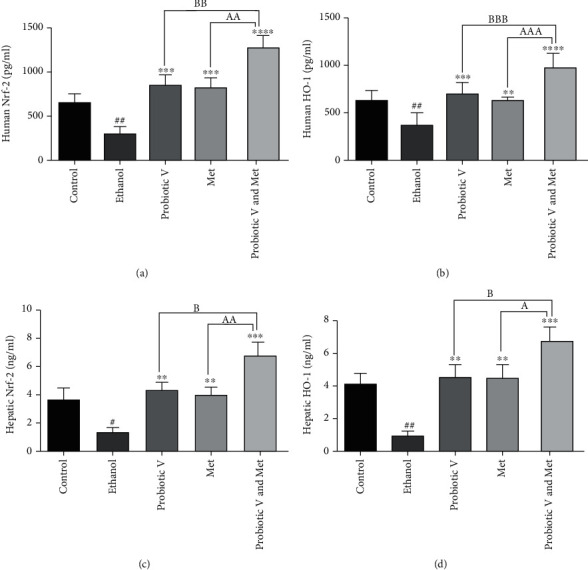
Protein levels of (a) human Nrf-2, (b) human HO-1, (c) hepatic Nrf-2, and (d) hepatic HO-1 analyzed through ELISA. Probiotic V and Met alone or in combination helps in regulating the production of antioxidants in HepG2 cells and Wistar rats. Values are expressed as mean ± SD of three individual experiments (HepG2 cells) and six rats. Statistical significance was assessed by one-way ANOVA followed by Tukey post hoc test. Statistical analysis: ^#^*p* < 0.05 and ^##^*p* < 0.01 compared with control group; ^∗∗^*p* < 0.01, ^∗∗∗^*p* < 0.0001, and ^∗∗∗∗^*p* < 0.0001 compared with ethanol group; ^A,B^*p* < 0.05, ^AA,BB^*p* < 0.01, ^AAA,BBB^*p* < 0.001, and ^AAAA,BBBB^*p* < 0.0001 compared with probiotic V and Met combinatorial group.

As shown in Figure 12, decreased levels of antioxidant proteins, i.e., Nrf-2 and HO-1, were observed in the ethanol group when compared with a control group. However, combined treatment of probiotic V and Met showed increased levels of Nrf-2 and HO-1 compared to the ethanol group, which was more significantly reduced as compared to either individual treatment of probiotic V or Met. Thus, combinatorial treatment of probiotic V and Met helps in reducing the production of Nrf-2 and HO-1 protein levels in both the *in vitro* and *in vivo* models of ethanol-induced hepatic injury.

### 3.12. Combination of Probiotic V and Met Regulates the Polarization of Macrophages on Ethanol-Exposed RAW 264.7 Cell Line

**Figure 13:**
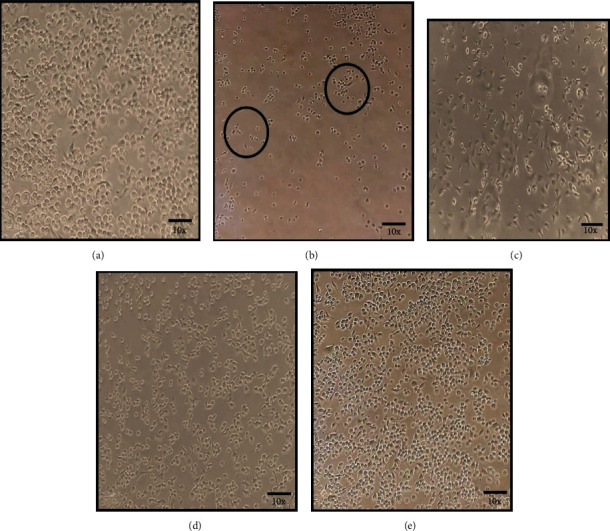
Microscopic images of RAW 264.7 cells treated with (a) control, (b) 100 mM ethanol control, (c) 100 mM ethanol+1 mM Met, (d) 100 mM ethanol+10 *μ*l/ml probiotic V, and (e) 100 mM ethanol+10 *μ*l/ml probiotic V and 1 mM Met combination. The above figures are representative of 10x objective images of three different experiments. RAW 264.7 cells treated with ethanol alone or along with probiotic V and Met alone or in combination for 48 h. Ethanol-exposed RAW 264.7 cells showed increased cellular distortion. Co-treatment of ethanol with probiotic V and Met in combination showed improved cellular integrity with round-shaped morphology, similar to the control cells as compared to the ethanol-exposed RAW 264.7 cells. However, differences were also observed between the individual treatment of either probiotic V and Met alone when compared with the combination treatment (i.e., probiotic V and Met).

**Figure 14:**
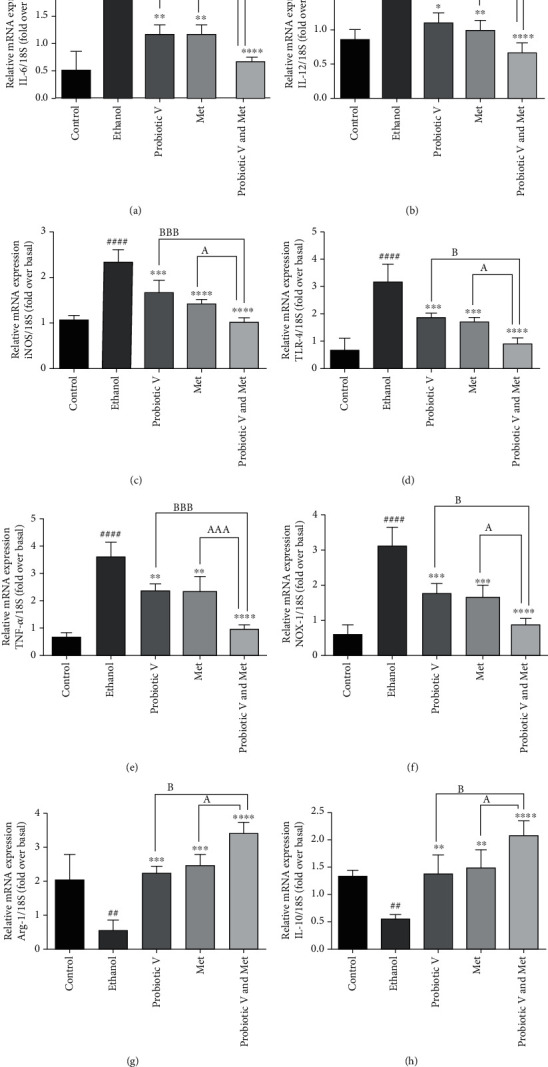
qRT-PCR analysis of (a) IL-6, (b) IL-12, (c) iNOS, (d) TLR-4, (e) TNF-*α*, (f) NOX-1, (g) Arginase-1, and (h) IL-10 genes from ethanol-exposed RAW 264.7 cells along with probiotic V and Met alone or together as probiotic V and Met combination for 48 h. The gene expression levels were calculated after stabilizing against 18S rRNA in every sample and are represented as relative mRNA expression units. Values represent the mean ± SD of three individual experiments. Statistical significance was assessed by one-way ANOVA followed by Tukey *post hoc* test. Statistical analysis: ^##^*p* < 0.01, ^###^*p* < 0.001, and ^####^*p* < 0.0001 compared with control group; ^∗^*p* < 0.05, ^∗∗^*p* < 0.01, ^∗∗∗^*p* < 0.001, and ^∗∗∗∗^*p* < 0.0001 compared with ethanol group; ^A,B^*p* < 0.05, ^AA,BB^*p* < 0.01, and ^AAA,BBB^*p* < 0.001 compared with probiotic V and Met combinatorial group.

Macrophages play an important role in fighting against pathogens, tissue restoration, and inflammation rectification. Chronic alcohol consumption causes macrophage function dysregulation [32]. Constant with these results, our data also validated that ethanol-exposed RAW 264.7 cells showed upregulated gene expression of markers of M1 phenotype compared to control cells. The untreated control RAW 264.7 cells are round and small in morphology, but ethanol treatment changed their shape and cells demonstrated synaptic morphology as seen by their elongated, pointed ends. Cotreatment of probiotic V and Met individually and in combination along with ethanol showed improved morphology when compared with ethanol-exposed RAW 264.7 cells (Figure 13). Moreover, combinatorial treatment of probiotic V and Met showed more significant morphological changes as compared to the individual treatment of either probiotic V or Met. At the molecular level, probiotic V or Met alone and in combination with probiotic V observed decreased expression levels of markers of M1 phenotype like IL-6, IL-12, TNF-*α*, TLR-4, iNOS, and NADPH oxidase 1 (NOX-1) and increased expression levels of M2 phenotypes, such as IL-10 and Arginase-1 as compared to ethanol-induced RAW 264.7 cells (Figure 14). However, combinatorial treatment of probiotic V and Met also showed significant differences in M1 and M2 phenotype macrophage expression levels when compared with the individual treatment of probiotic V or Met.

## 4. Discussion

Consumption of alcohol is considered to be the only major cause for the development of liver injury. Excessive alcohol consumption streams down through the portal vein and reaches the liver and develops toxicity, leading to oxidative stress and lipid accumulation in hepatocytes [33]. Therefore, oxidative stress and inflammation are considered to be the two important players in the progression of ethanol-induced liver injury [34, 35]. To study ethanol-induced hepatic injury, HepG2 cells are widely used as an *in vitro* study model [36]. One study also displayed that metformin is capable of regulating the microenvironment of macrophage polarization and prevent the proliferation of HepG2 cells [37]. Also, the liver is considered to be the major organ for ethanol metabolism. In the liver, ethanol metabolizes to acetaldehyde and forms adducts with proteins and DNA, later inducing various allosteric changes resulting in loss of its function [38]. Several reports displayed that chronic alcohol consumption resulted in histopathological modifications and altered the activity of hepatic enzymes and lipid profiles [39]. ALT, AST, and ALP are vital biochemical markers indicative of liver injury [40]. The present study showed a significant increase in serum transaminases (ALT and AST) and ALP levels in the ethanol-fed group. The elevated serum levels can be due to the outflow of cellular enzymes into the bloodstream. Clinically, fatty liver change is easily observed for diagnostic purposes, but liver histology is necessary to make a definitive diagnosis of the liver to see the extent of liver injury. Our results are in accordance with the above reports depicting increased ALT, AST, and ALP levels with the dramatic increase in the lipid deposits that occurred in the central lobular portions, hepatocyte swelling with the disorderly arrangement in the ethanol-fed rats, representing the pathologic alteration in the morphology of the liver. According to our protocol, these changes are indicative of ethanol-induced liver injury in a rat model. Currently, there are no FDA-approved therapies available to treat or prevent alcoholic liver injury. Abstinence from drinking alcohol is the only way to curtail the development and progression of alcoholic liver injury. Currently, available therapy to treat an ethanol-induced liver injury is single, and the therapeutic effect is not ideal [41]. Two such individual known therapies are probiotics and Met which protect against alcoholic liver injury [42, 43]. However, combined treatment of effective therapies may provide different links and targets to treat or prevent ethanol-induced liver injury. This is the first report, to our knowledge, which provides a piece of strong evidence indicating that the novel synergistic effect of probiotic V in combination with Met could significantly prevent the development of ethanol-induced liver injury.

Zhu et al. (2014) found that metformin at a dose of 200 mg/kg/day attenuated the liver injury caused by chronic ethanol exposure [44]. Also, it was reported that liver fibrosis was reduced by the oral administration of probiotic lactic acid bacteria at a dose of 10^9^ CFU/ml in an in vivo model [45]. Therefore, to explore whether the lower dose of probiotic V and Met shows a protective effect against ethanol-induced liver injury when administered alone and in combination, we chose 10^8^ CFU/ml/day probiotic V and 75 mg/kg Met as a combinatorial treatment. In the current study, we found that the combined treatment of probiotic V and Met could effectively improve the increased serum ALT, AST, and ALP levels in ethanol-fed rats. The above results demonstrated that the combined treatment of probiotic V and Met at a lower dose had an improved protective effect against ethanol-induced liver toxicity.

Ethanol-induced ROS production plays a key role in the progression of ALD and limits the cytoprotective gene expression [46]. Therefore, the amelioration of ROS generation by the antioxidant resistance network could help preserve the intracellular redox homeostasis. The current data prove that ethanol-exposed HepG2 cells showed increased ROS accumulation, which was rescued by the combined therapy with probiotic V and Met more significantly as compared with the individual treatment of probiotic V and Met, as evidenced by lower intracellular ROS accumulation. Generation of ROS through ethanol metabolism via CYP2E1 leads to lipid peroxidation and the production of reactive aldehydes like 4-hydroxyl nonenal (4-HNE), which further form protein and DNA adducts [47]. In the ER, the accumulation of protein adducts causes ER fragmentation, therefore leading to ER stress [48]. Our results are in accordance with the reports suggesting that the Met and probiotic *L*. *acidophilus* can ameliorate ER stress in HepG2 cells [49] and HT-29 cells as well as *in vivo* [50] by decreasing the expression of CHOP and Grp78. In the present study, probiotic V and Met alone in ethanol-exposed HepG2 cells showed decreased mRNA expression of ER stress markers like CHOP and Grp78 but the combinatorial treatment of probiotic V and Met showed a more significant decrease as compared to the individual treatment of probiotic V or Met suggesting that probiotic V and Met in combination are capable of preventing ER stress.

Chronic alcohol consumption exerts a detrimental effect on lipid metabolism in the liver resulting in hepatic steatosis [51]. During the hepatic injury, lipogenic enzymes like HSL and PPAR-*γ* play a crucial role in lipid metabolism. In the liver, increased lipogenesis was observed with the hepatic overexpression of PPAR-*γ* [52]. HSL transcripts are found to be elevated in livers overexpressing PPAR-*γ* [53]. Our report observed reduced lipogenesis in the HepG2 cells (as seen by reduced lipid droplets staining by Oil red O) and ethanol-fed rats treated with probiotic V and Met compared to the ethanol group. The current results are supported by Choi et al. (2020), where they demonstrated that probiotic *L*. *plantarum* administration reduced the hepatic weight and the mRNA expression levels of lipogenic genes PPAR-*γ* and HSL [54]. Also, current results demonstrated that probiotic V and Met in combination or alone administration suppressed the inhibition of AMPK levels induced by ethanol, which is known as a critical regulator of lipid metabolism. Oppositely, activation of AMPK inhibits the expression of transcription factor, i.e., SREBP-1c (also a key regulator of lipid metabolism), as observed in the combinatorial treatment of probiotic V and Met. Consistent with the altered expression of SREBP-1c, it also decreases the expression of downstream lipogenic enzymes like ACC and FAS compared to ethanol-treated groups as well as either individual treated group. Our results are in accordance to Zhu et al. (2014) and Zhang et al. (2015), where the administration of individual treatment of Met and *Lactobacillus rhamnosus* GG activated AMPK and thereby downregulated the expression levels of SREBP-1c, ACC, and FAS in ethanol-induced liver injury model [44, 55].

Another important marker of alcohol-induced liver injury is demonstrated by the increased levels of TG, which is indicative of hepatic steatosis. Fatty acid oxidation and tricarboxylic acid (TCA) circulation get disturbed due to excessive alcohol intake which affects the lipid metabolism, causing increased accumulation of TG in the liver and therefore elevates the serum levels of TG [56]. Feeding the rats with probiotic V and Met showed diffused lipid accumulation with overall better morphology along with the decreased content of hepatic TG and TC as compared to the individual treatment of probiotic V and Met. The above results are in accordance with Shavakhi et al., where combined treatment of Met and probiotics can reduce the serum levels of TC and TG in NASH [57]. All these factors contribute to the fact that probiotic V and Met administration helps in regulating the lipid metabolism in the liver, thereby preventing ethanol-induced lipogenesis. Hence, probiotic V and Met co-administration to ethanol-exposed HepG2 cells proved to be efficient in regulating lipid accumulation and prevent lipid peroxidation.

Ethanol-induced liver injury is linked to an increased level of nitrative stress. In a mouse model, it was observed that the ethanol-fed group was having an increased mRNA expression of ROS-generating enzymes like iNOS by 3.6 fold [58]. iNOS expression was increased in HepG2 cells and is shown to be required for ALD [59]. Our results are in agreement with the literature that suggests the role of Met [60], and probiotic *Lactobacillus* [61] can prevent liver injury by reducing the production of iNOS and nitric oxide in the liver. The present study also showed that gene expression level of iNOS was decreased by treating individually with probiotic V and Met to ethanol-exposed HepG2 cells, which had upregulated in ethanol-exposed HepG2 cells. However, in the presence of ethanol, combined treatment of probiotic V and Met significantly reduces the expression levels of iNOS as compared to either individual treatment of probiotic V or Met.

Alterations in the development of ROS/RNS (reactive nitrogen species) result in the onset of oxidative stress. Acetaldehyde and MDA are produced due to oxidation of the alcohol during its metabolism, and an increased level of MDA is an oxidative stress biomarker [62]. Met along with probiotic showed an inhibitory effect by alleviating the oxidative stress in colorectal cancer (CRC) and type 2 diabetes [19]. Our results explain that ethanol-exposed HepG2 cells persist with upregulated MDA levels, whereas the elevation in MDA levels was significantly prevented by the combinatorial treatment of probiotic V and Met as compared to the individual treatment of probiotic V or Met demonstrating the combined administration of probiotic V and Met is potentially effective in preventing ethanol-induced oxidative stress. This possibly explains the innate antioxidant capacity of probiotic V and Met in preventing ethanol-induced oxidative stress, ER stress, ROS generation, and/or lipid peroxidation.

Persistent ROS results in inflammation which leads to a major number of illnesses in humans. Cells that are impaired due to oxidative stress induce inflammation upon alcohol ingestion [63]. Chronic alcohol consumption induces CYP2E1, which plays a pivotal part in ROS production [64]. Studies have shown increased expression of CYP2E1 induced by 100 mM ethanol resulting in alcohol-induced liver injury [65]. The extant study indicates that cotreatment of a probiotic V and Met in ethanol-exposed HepG2 cells and ethanol-fed rats showed reduced mRNA expression levels of CYP2E1 as compared to the ethanol group as well as the individual treatment of either probiotic V and Met. HO-1 plays a significant role in the defense mechanism against oxidative damage [66], and its expression is noticeably reduced in hepatocytes treated with ethanol [67]. However, in the present study, the ethanol led to the decline expression of HO-1 cytoprotective enzyme, which was prevented by probiotic V and Met cotreatment in ethanol-exposed HepG2 cells as well as ethanol-induced rat model. Also, Nrf-2 binds to an upstream promoter region of ARE (antioxidant response elements) genes including HO-1 [48]. In hepatic tissues, studies proved that the upregulated activity of Nrf-2 and HO-1 is extremely hepatoprotective during oxidative stress [68, 69]. The present result indicates that probiotic V and Met treatment significantly elevates the Nrf-2 activity in ethanol-exposed HepG2 cells as well as ethanol-fed rats. This proves that probiotic V and Met in combination helps in significantly reducing oxidative stress caused by CYP2E1 through elevating the levels of HO-1 and Nrf-2 in HepG2 cells as well as rat models. However, the combined treatment of probiotic V and Met showed a significant difference in the expression levels of HO-1 and Nrf-2 as compared to the individual treatment of probiotic V or Met.

TNF-*α* cytotoxicity and IL-1*β* secretion are known to be increased by ethanol exposure in the HepG2 cells and in rat primary hepatocytes [24, 58]. Met along with probiotic showed inhibitory effect by downregulating the expression of the inflammatory cytokines like TNF-*α* in colorectal cancer (CRC) and type 2 diabetes [19]. Our results are suggestive of chronic alcohol exposure-mediated upregulation of the expression levels of TNF-*α* and IL-1*β*, which were downregulated more significantly by cotreating probiotic V and Met in combination as compared to the individual treatment of either agent. TNF-*α* also downregulated by the production of IL-10, which otherwise is decreased in HepG2 cells challenged with 100 mM ethanol [70] consistent with the current study. We, in our study report, decrease in the mRNA expression levels of IL-10 by ethanol which is alleviated by a combination of probiotic V and Met in HepG2 cells as compared to ethanol-exposed cells. Significantly, in the presence of probiotic V and Met, ethanol-exposed HepG2 cells showed more elevated mRNA expression levels of IL-10 than the individual treatment of probiotic V or Met.

TLR-4 plays an important role in the development of ALD. In HepG2 cells, increased TLR-4 expression was induced by 100 mM ethanol for 20 h [70]. In the NASH model, treatment with Met or probiotic (*L*. *reuteri*) and antibiotic metronidazole revealed modest improvement of the inflammatory pathway: LPS/TLR-4/NF-*κ*B/TNF-*α* [20]. The current study showed an increase in TLR-4 mRNA expression levels in ethanol-exposed HepG2 cells, which was downregulated by cotreating probiotic V and Met in ethanol-exposed HepG2 cells as well as ethanol-fed rats. However, in the presence of ethanol, combined treatment of probiotic V and Met significantly reduced the expression levels of TLR-4 as compared to the individual treatment of probiotic V or Met.

In the liver, macrophages also play an acute role in the progression of alcohol-induced inflammation and the accumulation of infiltrating macrophages [71]. Excessive consumption of alcohol leads to the activation of Kupffer cells, which act upon a response to increased intestinal translocation of LPS through the CD14/TLR-4 receptor complex and contribute to ALD progression [72]. Studies have shown increased release of TNF-*α* and IL-6 in RAW 264.7 cells stimulated by 100 mM ethanol for 24 h and that Met-induced RAW 264.7 macrophage towards the M2 phenotype [3]. Given the role of alcohol in activating macrophages to produce pro-inflammatory cytokines [73], it was required to identify if cotreatment of a probiotic V and Met with ethanol-induced RAW 264.7 cells could promote the expression of the anti-inflammatory cytokine. Our study evinced that ethanol exposure to RAW 264.7cells upregulated the gene expression of M1 phenotypic markers compared to control cells, which was prevented by cotreatment of probiotic V and Met as well as their individual treatment along with ethanol, as suggested by decreased expression levels of M1 phenotype markers like IL-6, IL-12, iNOS, TLR-4, TNF- *α*, and NOX-1. Our study observed the combined treatment of probiotic V and Met also upregulated the expression levels of M2 phenotypic markers like Arginase-1 and IL-10 compared to ethanol-treated RAW 264.7 cells. This study additionally concludes that combinatorial treatment of probiotic V and Met may serve as a preventive target for ALD progression as it helps in translating the pro-inflammatory M1 phenotype macrophages (tissue-damaging infiltrative macrophages) to M2 phenotype macrophages (anti-inflammatory tissue restorative cells) more significantly compared to the probiotic V or Met-unaided groups.

In the current study, the combination of probiotic V and Met helped Met in potentiating its antioxidative and anti-inflammatory effects. Our findings indicate that probiotic V and Met in combination prevent ethanol-induced cytotoxicity by inhibiting oxidative stress and ROS generation and protect human HepG2 cells and the *in vivo* hepatic injury by CYP2E1 inhibition. The reduced expression levels of CYP2E1 induced by probiotic V and Met are associated with the increased Nrf-2 translocation as the Nrf-2 translocation and HO-1 activation accelerate the heme decomposition, resulting in a reduced CYP2E1 protein level [74]. We hypothesize that probiotic V and Met in combination may possibly inhibit LPS/TLR-4 signaling and downregulate the phosphorylation of NF-*κ*B or MAPK, triggering the production of inflammatory mediators like TNF-*α*, IL-1*β*, and IL-6, thereby preventing the ethanol-induced hepatic injury.

## 5. Conclusion

**Figure 15:**
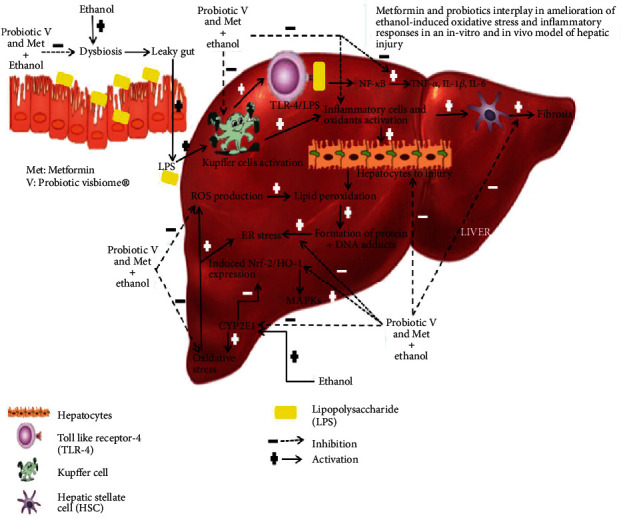
Possible mechanism contributing to the combined protecting effect of probiotic V and Met against ethanol-induced hepatic injury.

Results showed in the current study lead to numerous conclusions showing the contribution in the improvement of direct therapies by the combined presence of probiotic V and Met. Results from the present study suggested that co-administration of probiotic V and Met with ethanol exposure was efficient in improving the cellular injury and liver histology in the ethanol-exposed HepG2 cells, RAW 264.7 cells, and ethanol-fed rats. Also, co-administration of probiotic V and Met with ethanol considerably reduced the inflammatory response and lipid accumulation, upregulated antioxidant levels, and regulated the lipid metabolism in an in vitro and in vivo model of ethanol-induced hepatic injury. The present study inferred the influence of combined treatment of probiotic V and Met in the presence of ethanol at the cytokine expression as well as protein levels. Therefore, reduced levels of ROS and oxidative stress as well as inflammatory markers might be the potential targets for the preventive effects presented by probiotic V and Met in combination. The activation of the MAPK/Nrf-2/HO-1 signaling pathway might be a possible mechanism contributing to the combined protecting effect of probiotic V and Met against ethanol-induced HepG2 cytotoxicity and hepatic injury (Figure 15). Among alcohol users, this study has clinical inference with respect to ethanol-induced hepatic injury.

## Figures and Tables

**Table 1:** The list of Homo sapiens primers used for quantification of mRNA expression levels in qRT-PCR.

Sr. No.	Name of the gene	Forward primer sequence	Reverse primer sequence
1.	18S	ACGGAAGGGCACCACCAGGA	CACCACCACCCACGGAATCG
2.	iNOS	CCCTTCCGAAGTTTCTGGCAGCAGC	GGCTGTCAGAGAGCCTCGTGGCTTTGG
3.	CYP2E1	AGGGTACCATGTCTGCCCTCGGAGTGA	ACAATTTGAAAGCTTGTTTGAAAGCGG
4.	TNF-*α*	CCCTCACACTCAGATCATCTTCT	GCTACGACGTGGGCTACAG
5.	IL-10	ACTGCTAACCGACTCCTTA	TAAGGAGTCGGTTAGCAGT
6.	HO-1	AAGCCGAGAATGCTGAGTTCA	CGGGTGTAGATATGGTACAAGGA
7.	IL-6	GACAACTTTGGCATTGTGG	ATGCAGGGATGATGTTCTG
8.	CHOP	GAAAGCAGAAACCGGTCCAAT	GGATGAGATATAGGTGCCCCC
9.	Grp78	GAAACTGCCGAGGCGTAT	ATGTTCTTCTCTCCCTCTCTCTTA
10.	TLR-4	GATTGCTCAGACATGGCAGTTTC	CACTCGAGGTAGGTGTTTCTGCT AA
11.	Nrf-2	TGCCCCTGGAAGTGTCAAACA	CAACAGGGAGGTTAATGATTT
12.	PPAR-*γ*	GGCTTCATGACAAGGGAGTTTC	AACTCAAACTTGGGCTCCATAAAG
13.	HSL	GTGCAAAGACGGAGGACCACTCCA	GACGTCTCGGAGTTTCCCCTCAG
14.	AMPK	GGGTGAAGATCGGACACTACGT	TTGATGTTCAATCTTCACTTTG
15.	SREBP-1c	CGGCGCGGAAGCTGT	TGCAATCCATGGCTCCGT
16.	ACC	CTGCTCGTGGATGAACCAGAC	GTCAGCCATCGCCCGAGC
17.	FAS	ACAGGGACAACCTGGAGTTCT	CTGTGGTCCCACTTGATGAGT

**Table 2:** The list of Mus musculus primers used for quantification of mRNA expression levels in qRT-PCR.

Sr. No.	Name of the gene	Forward primer sequence	Reverse primer sequence
1.	18S	GTAACCCGTTGAACCCCATT	CCATCCAATCGGTAGTAGCG
2.	Arginase-1	GCTGTCTTCCCAAGAGTTGGG	ATGGAAGAGACCTTCAGCTAC
3.	IL-12	GAAAGACCCTGACCATCACT	CCTTCTCTGCAGACAGAGAC
4.	NOX-1	TTAAACAAGAAGGAACTACT	CTAATAAACGTCTGCTGC
5.	IL-6	GAGGATACCACTCCCAACAGACC	AAGTGCATCATCGTTGTTCATACA
6.	TNF-*α*	GGTGCCTATGTCTCAGCCTCTT	CCATAGAACTGATGAGAGGGAG
7.	TLR-4	CCTGATGACATTCCTTCTTCAAC	TTGTTTCAATTTCACACCTGGATAAA
8.	iNOS	TCACTGGGACAGCACAGAAT	TGTGTCTGCAGATGTGCTGA
9.	IL-10	CGGGAAGACAATAACTGCACCC	CGGTTAGCAGTATGTTGTCCAGC

**Table 3:** The list of Rattus norvegicus primers used for quantification of mRNA expression levels in qRT-PCR.

Sr. No.	Name of the gene	Forward primer sequence	Reverse primer sequence
1.	18S	GTTGGTTTTCGGAACTGAGGC	GTCGGCATCGTTTATGGTCG
2.	CYP2E1	TCAATCTCT GGACCCCAACTG	GCGCTCTGCACTGTGCTTT
3.	TLR-4	TTGAAGACAAGGCATGGCATGC	TCTCCCAAGATCAACCGATG
4.	TNF-*α*	TCTCATTCCTGCTCGTGGCG	GGTGAGGAGCACGTAGTCGG
5.	IL-6	TTGACAGCCACTGCCTTCCC	CGGAACTCCAGAAGACCAGAGC
6.	HO-1	CAAATCCCACCTTGAACACA	CGACTGACTAATGGCAGCAG
7.	Nrf-2	CAGAGTTTCTTCGCCAGAGG	TGAGTGTGAGGACCCATCG
8.	PPAR-*γ*	ACGATCTGCCTGAGGTCTGT	CATCGAGGACATCCAAGACA
9.	HSL	AGTGAAAAACCCGCGGACC	TTCATCCTTCTGCCCCCTAC
10.	AMPK	GCTGTGGATCGCCAAATTAT	GCATCAGCAGAGTGGCAATA
11.	SREBP-1c	TCTGCCTTGATGAAGTGTGG	AGCAGCCCCTAGAACAAACA
12.	ACC	CCTTCTTCTACTGGCGACTGAG	TAAGCCTTCACTGTGCCTTCC
13.	FAS	CCTCAGTCCTGTTATCACCCGA	GCTGAATACGACCACGCACTA
14.	IL-10	TGCCTTCAGTCAAGTGAAGAC	AAACTCATTCATGGCCTTGTA

**Table 4:** Effect of probiotic V and Met on the viability of HepG2 cells. The average percentage of cell viability after respective treatment was analyzed through MTT assay. Values represent the mean ± SD of three individual experiments. Statistical significance was assessed by one-way ANOVA followed by Tukey post hoc test. Statistical analysis: ^####^*p* < 0.0001 compared with control untreated group; ^∗∗^*p* < 0.01, ^∗∗∗^*p* < 0.001, and ^∗∗∗∗^*p* < 0.0001 compared with ethanol alone; ns stands for nonsignificant.

Groups	Cell viability (%)	
	Control	Ethanol
Untreated	95.7	64.6^####^
Probiotic V (10 *μ*l/ml)	95.5	81.9^∗∗∗^
Probiotic V (50 *μ*l/ml)	95.3	83.1^∗∗∗^
Probiotic V (100 *μ*l/ml)	95.3	85.5^∗∗∗∗^
Met (1 mM)	94.9	82.7^∗∗∗∗^
Met (2 mM)	93.5	81.6^∗∗∗^
Met (3 mM)	87.5	67.9^ns^
Probiotic V (10 *μ*l/ml)+Met (1 mM)	95.5	86.8
Probiotic V (10 *μ*l/ml)+Met (2 mM)	95.3	81.2
Probiotic V (10 *μ*l/ml)+Met (3 mM)	88.2	69.2
Probiotic V (50 *μ*l/ml)+Met (1 mM)	95.2	87.0
Probiotic V (50 *μ*l/ml)+Met (2 mM)	95.2	81.8
Probiotic V (50 *μ*l/ml)+Met (3 mM)	89.5	70.2
Probiotic V (100 *μ*l/ml)+Met (1 mM)	95.2	87.3
Probiotic V (100 *μ*l/ml)+Met (2 mM)	95.2	82.3
Probiotic V (100 *μ*l/ml)+Met (3 mM)	91.3	70.5

## Data Availability

The authors confirm that the data supporting the findings of this study are available within the article.

## Supplementary Materials

Supplementary Materials The effect of probiotic V and Met on the viability of RAW 264.7 murine macrophage cells.
