# Supplementary material for: Metformin and Probiotics Interplay in Amelioration of Ethanol-Induced Oxidative Stress and Inflammatory Response in an In Vitro and In Vivo Model of Hepatic Injury
Source: Mediators Inflamm. 2021 Apr 15;2021:6636152. doi: 10.1155/2021/6636152 (PMC8064785; doi:10.1155/2021/6636152)
Supplement: Supplementary Materials — The effect of probiotic V and Met on the viability of RAW 264.7 murine macrophage cells. [file 6636152.f1.docx]

**Supplementary Material 1**

**Method: Cell Viability Assay**

In the present *in-vitro* experiments, the RAW 264.7 cell line was treated with the suspension of bacterial lysates’ at final concentrations of 10, 50, and 100 μl/ml, corresponding, to 1, 5, and 10 mg (lyophilized bacterial mass) / ml. Cells were supplemented with a total of the bacterial lysate (10, 50, and 100 μl/ml) corresponding to 10^8^, 5 x 10^8^, and 10^9^ CFU/ ml in the culture medium. For each experiment, fresh bacterial lysates of probiotic V and Met were prepared for experimental studies.

The concentration of ethanol was determined based on the literature survey [1]. 3-(4,5-dimethylthiazol-2-yl) -2,5-diphenyltetrazolium bromide (MTT) assay was used to determine the cell viability as described by Kema *et al.* 2018 [1]. In brief, 2 x 10^4^ cells were seeded in a 96 well plate. After the cells attained the desired confluency, they were exposed to different concentrations of probiotic V (10, 50, 100 μl/ml), Met (1, 2, 3 mM), individually and in combinatorial doses (10 µl/ml probiotic V with 1 mM, 2 mM, and 3 mM Met; 50 µl/ml probiotic V with 1 mM, 2 mM, and 3 mM Met; and 100 µl/ml probiotic V with 1 mM, 2 mM, and 3 mM Met) in the presence and absence of 100 mM ethanol for 48 h. After 48 h treatment, each well was incubated with 10 μl of MTT solution (0.45 mg/ml: final concentration) and kept in the dark at 37°C in a 5% CO_2_ atmosphere for 3 h. After the incubation period was over, to each well 100 μl of dimethyl sulfoxide (DMSO) was added to solvate the formazan crystals. To determine the cell viability, absorbance was measured at 570 nm for each sample and calculated as in Equation 1 as follows:

Cell viability (%) = As/Ac × 100 (1)

As and Ac represent the absorbance of sample treatment and control respectively.

**Result:** Toxicity of probiotic V (10, 50, and 100 μl/ml) and Met (1, 2, and 3 mM) individually on RAW 264.7 cell was assessed in the presence and absence of 100 mM ethanol (Supplementary Figure 1A). The effect of the combinatorial doses of probiotic V and Met (10 µl/ml probiotic V with 1, 2, and 3 mM Met; 50 µl/ml probiotic V with 1, 2, and 3 mM Met; and 100 µl/ml probiotic V with 1, 2, and 3 mM Met) also was assessed as a measure of cell viability on RAW 264.7 cells treated in the presence and absence of 100 mM ethanol (Supplementary Figure 1B and 1C).

Neither the individual treatment with probiotic V nor with Met at different concentrations showed significant cell death of RAW 264.7 cells in the absence of 100 mM ethanol. However, there was a slight reduction observed in the viability of the cells treated with 3mM Met alone. Treatment with 100 mM ethanol on RAW 264.7 cells induced cell death and therefore only 65.2% cell viability was observed after 48 h of 100 mM ethanol treatment as compared to the untreated cells (i.e. 95.9%). To see, if probiotic V could prevent the ethanol-induced toxicity, RAW 264.7 cells were treated with probiotic V in the presence of 100 mM ethanol. Our results indicate that probiotic V treatment at different concentrations (10, 50, and 100 μl/ml) show a dose-dependent slight increase in cell viability (83.73%, 83.81%, and 85.41% respectively) when compared to ethanol-exposed RAW 264.7 cells at 48 h. The effect of Met at various concentrations in the presence of 100 mM ethanol also was assessed as a measure of cell viability of RAW 264.7 cells. The viability of cells exposed to 100 mM ethanol in the presence of Met alone showed a dose-dependent decrease in cell viability (83.95%, 79.75%, and 68.3%, respectively) when compared to ethanol-exposed RAW 264.7 cells at 48 h, which is suggestive of the toxicity of Met at higher concentration.

As the treatment with probiotic V alone showed a dose-dependent increase, and the treatment with Met alone showed a dose-dependent decrease in the viability, we checked if the combination of probiotic V and Met could work in synergy to improve the cell viability of RAW 264.7 cells when treated in the presence of 100 mM ethanol. The combinatorial doses of probiotic V and Met did not significantly affect the viability of RAW 264.7 cells treated in the absence of 100 mM ethanol across all the different combinations. However, we observed that probiotic V treatment at various concentrations freed the toxicity of 3mM Met, and improved the cell viability as compared to individual treatment of Met (3 mM) in presence of ethanol. This suggests that probiotic V can improve the efficacy of metformin by reducing its toxicity at a higher concentration. Also, the viability of the cells treated with 100 mM ethanol in the presence of different combinations of probiotic V and Met was improved as compared to ethanol-treated RAW 264.7 cells in the absence of probiotic V and Met, which suggests the beneficial role of probiotic V and Met as combinatorial treatment. In accordance to result obtained from HepG2 cells, a similar significant difference in the cell viability was observed with a combination of 100 μl/ml probiotic V and 1 mM Met (85.86%) in RAW 264.7 cells, but this difference was not significantly different when compared to the cells treated with only 100 μl/ml probiotic V in the presence of ethanol (85.41%). As the main aim of our study is to see if probiotic V and Met could work in synergy to prevent the ethanol-induced toxicity of RAW 264.7 cells, we chose the combination of 10 μl/ml probiotic V and 1mM Met, because this combination significantly improved the cell viability of ethanol-exposed RAW 264.7 cells, compared to the individual treatments probiotic V or Met.

**Supplementary Table 1:** Effect of probiotic V and Met on the viability of RAW 264.7 cells. The average percentage of cell viability after respective treatment was analyzed through MTT assay. Values represent the mean ± SD of three individual experiments.

|  | **Cell viability (%)** | |
| --- | --- | --- |
|  | **Control** | **Ethanol** |
| Untreated | 95.9 | 65.2 |
| Probiotic V (10 μl/ml) | 95.5 | 83.73 |
| Probiotic V (50 μl/ml) | 95 | 83.81 |
| Probiotic V (100 μl/ml) | 94.83 | 85.41 |
| Met (1 mM) | 93.06 | 83.95 |
| Met (2 mM) | 92.39 | 79.75 |
| Met (3 mM) | 92.06 | 68.3 |
| Probiotic V (10 μl/ml) + Met (1 mM) | 95.23 | 85.43 |
| Probiotic V (10 μl/ml) + Met (2 mM) | 95.26 | 80.1 |
| Probiotic V (10 μl/ml) + Met (3 mM) | 94.8 | 68.97 |
| Probiotic V (50 μl/ml) + Met (1 mM) | 95.5 | 85.6 |
| Probiotic V (50 μl/ml) + Met (2 mM) | 95.46 | 80.73 |
| Probiotic V (50 μl/ml) + Met (3 mM) | 94.96 | 69.26 |
| Probiotic V (100 μl/ml) + Met (1 mM) | 95.23 | 85.86 |
| Probiotic V (100 μl/ml) + Met (2 mM) | 95.16 | 83.26 |
| Probiotic V (100 μl/ml) + Met (3 mM) | 94.76 | 70.01 |

**
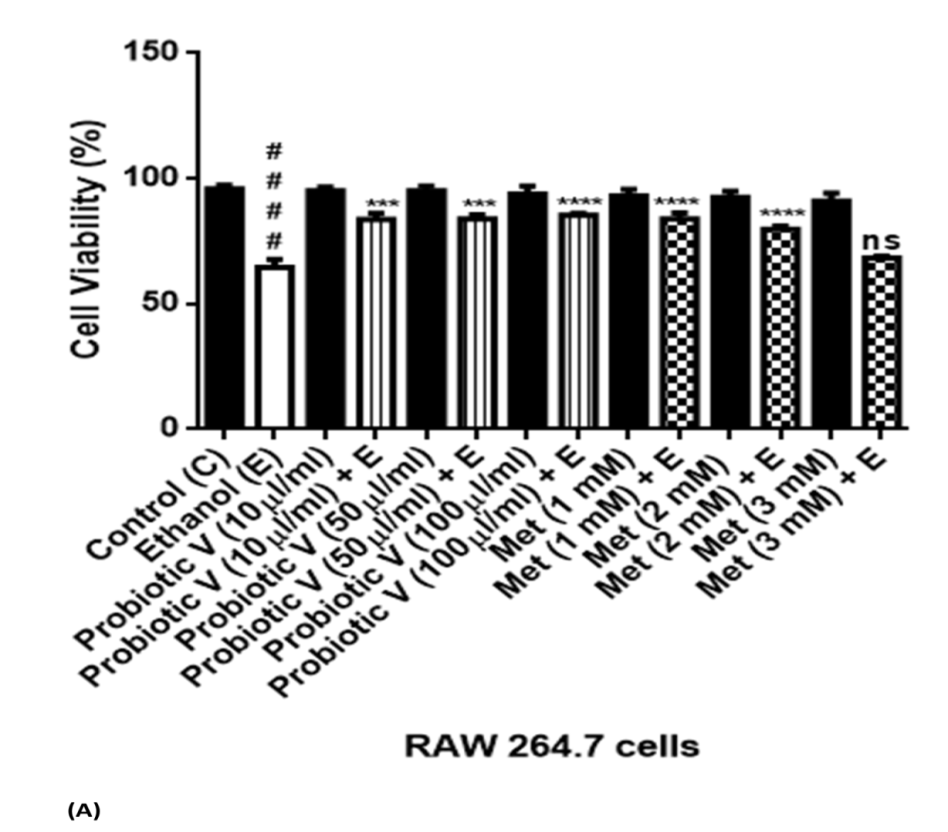
**

**
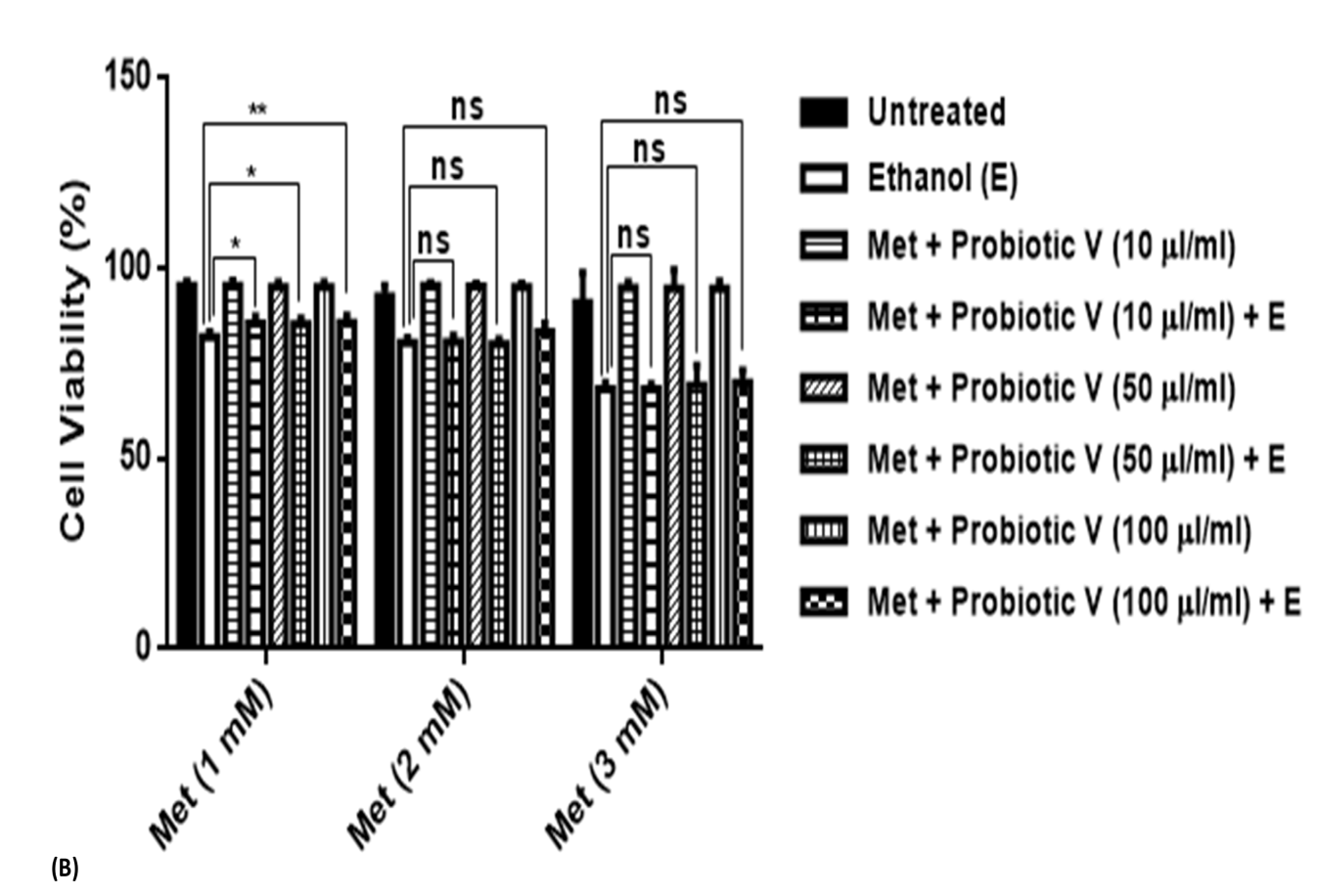
**

**
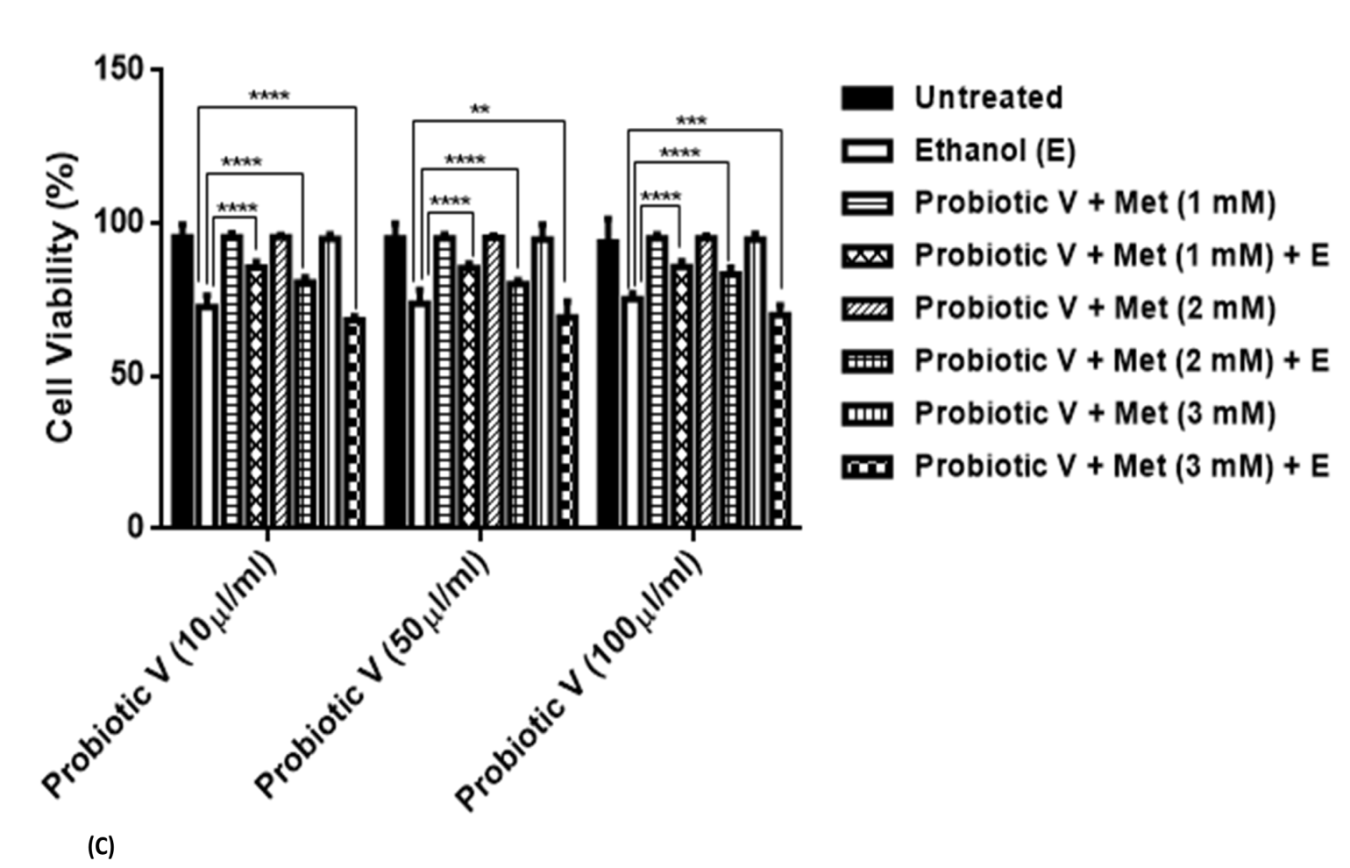
**

**Supplementary Figure 1:** Effect of probiotic V and Met on the viability of RAW 264.7 cells. (A) RAW 264.7 cells were exposed to 100 mM ethanol and co-treated individually with probiotic V (10, 50, and 100 µl/ ml) and Met (1, 2, and 3 mM) for 48 h. (B) RAW 264.7 cells were exposed to 100 mM ethanol and co-treated with probiotic V (10, 50 and 100 µl/ ml) and Met (1, 2, and 3 mM) in different combinations and compared with the individual doses of Met 1, 2, and 3 mM for 48 h. (C) RAW 264.7 cells were exposed to 100 mM ethanol and co-treated with probiotic V (10, 50 and 100 µl/ ml) and Met (1, 2, and 3 mM) in different combinations and compared with the individual doses of probiotic V (10, 50 and 100 µl/ ml) for 48 h. The average percentage of cell viability after respective treatment was analyzed through MTT assay. Values represent the mean ± SD of three individual experiments. Statistical significance was assessed by one-way ANOVA followed by Tukey-*post hoc* test. Statistical analysis: ^####^ p<0.0001 compared to control; *p<0.05, **p<0.01, ***p<0.001, and ****p<0.0001 compared to ethanol as well as individual treatment of probiotic V (10, 50 and 100 µl/ ml) and Met (1, 2 and 3 mM) in different combinations; ns stands for nonsignificant.

**Reference:**

1. Kema VH, Khan I, Kapur S, Mandal P. Evaluating the effect of diallyl sulfide on regulation of inflammatory mRNA expression in 3T3L1 adipocytes and RAW 264.7 macrophages during ethanol treatment. *Drug Chem Toxicol*. 2018;41(3):302-13;DOI:10.1080/01480545.2017.1405969.
